# Synaptic vesicle dynamic changes in a model of fragile X

**DOI:** 10.1186/s13229-016-0080-1

**Published:** 2016-03-01

**Authors:** Jantine A. C. Broek, Zhanmin Lin, H. Martijn de Gruiter, Heleen van ‘t Spijker, Elize D. Haasdijk, David Cox, Sureyya Ozcan, Gert W. A. van Cappellen, Adriaan B. Houtsmuller, Rob Willemsen, Chris I. de Zeeuw, Sabine Bahn

**Affiliations:** Cambridge Centre for Neuropsychiatric Research, Department of Chemical Engineering and Biotechnology, University of Cambridge, Cambridge, UK; Department of Neurosciences, Erasmus MC, Rotterdam, The Netherlands; Erasmus Optical Imaging Center, Erasmus MC, Rotterdam, The Netherlands; Department of Clinical Genetics, Erasmus MC, Rotterdam, The Netherlands; Netherlands Institute for Neurosciences, Royal Academy for Arts and Sciences, Amsterdam, The Netherlands

**Keywords:** Fragile X syndrome (FXS), Synaptic transmission, Mass spectrometry (MS), Quantitative live-cell imaging, Electron microscopy

## Abstract

**Background:**

Fragile X syndrome (FXS) is a single-gene disorder that is the most common heritable cause of intellectual disability and the most frequent monogenic cause of autism spectrum disorders (ASD). FXS is caused by an expansion of trinucleotide repeats in the promoter region of the fragile X mental retardation gene (*Fmr1*). This leads to a lack of fragile X mental retardation protein (FMRP), which regulates translation of a wide range of messenger RNAs (mRNAs). The extent of expression level alterations of synaptic proteins affected by FMRP loss and their consequences on synaptic dynamics in FXS has not been fully investigated.

**Methods:**

Here, we used an *Fmr1* knockout (KO) mouse model to investigate the molecular mechanisms underlying FXS by monitoring protein expression changes using shotgun label-free liquid-chromatography mass spectrometry (LC-MS^E^) in brain tissue and synaptosome fractions. FXS-associated candidate proteins were validated using selected reaction monitoring (SRM) in synaptosome fractions for targeted protein quantification. Furthermore, functional alterations in synaptic release and dynamics were evaluated using live-cell imaging, and interpretation of synaptic dynamics differences was investigated using electron microscopy.

**Results:**

Key findings relate to altered levels of proteins involved in GABA-signalling, especially in the cerebellum. Further exploration using microscopy studies found reduced synaptic vesicle unloading of hippocampal neurons and increased vesicle unloading in cerebellar neurons, which suggests a general decrease of synaptic transmission.

**Conclusions:**

Our findings suggest that FMRP is a regulator of synaptic vesicle dynamics, which supports the role of FMRP in presynaptic functions. Taken together, these studies provide novel insights into the molecular changes associated with FXS.

**Electronic supplementary material:**

The online version of this article (doi:10.1186/s13229-016-0080-1) contains supplementary material, which is available to authorized users.

## Background

Fragile X syndrome (FXS, OMIM: #300624) is a single-gene disorder causing a heritable form of mental impairment (WHO, 1996), with a prevalence in males ranging from 1/4000 to 1/5161 [1, 2]. Core cognitive deficits found in FXS-patients include problems with short-term and working memory, executive function deficits and mathematical and visuospatial difficulties [3]. Furthermore, 60–74 % of male FXS patients meet the criteria for autism spectrum disorder (ASD), and 2–6 % of ASD patients are identified to suffer from FXS [4, 5]. In spite of the clear monogenetic cause of FXS, the cellular consequences of fragile X mental retardation protein (FMRP) depletion are widespread and remain poorly understood. FMRP is known for its role in messenger RNA (mRNA) binding, transport activity and subsequent translation regulation upon metabotropic glutamate receptor 5 (mGluR5) stimulation [6, 7]. Synaptic dysfunction would be expected both in the pre- and postsynaptic regions. The systems found to be affected at the presynapse include synaptic vesicles [8] and plasticity via short-term depression [9, 10]. Despite the indication of synaptic differences in the pathogenesis of FXS, the underlying mechanisms of synaptic dysfunction in FXS remain to be investigated.

**Fig. 1:**
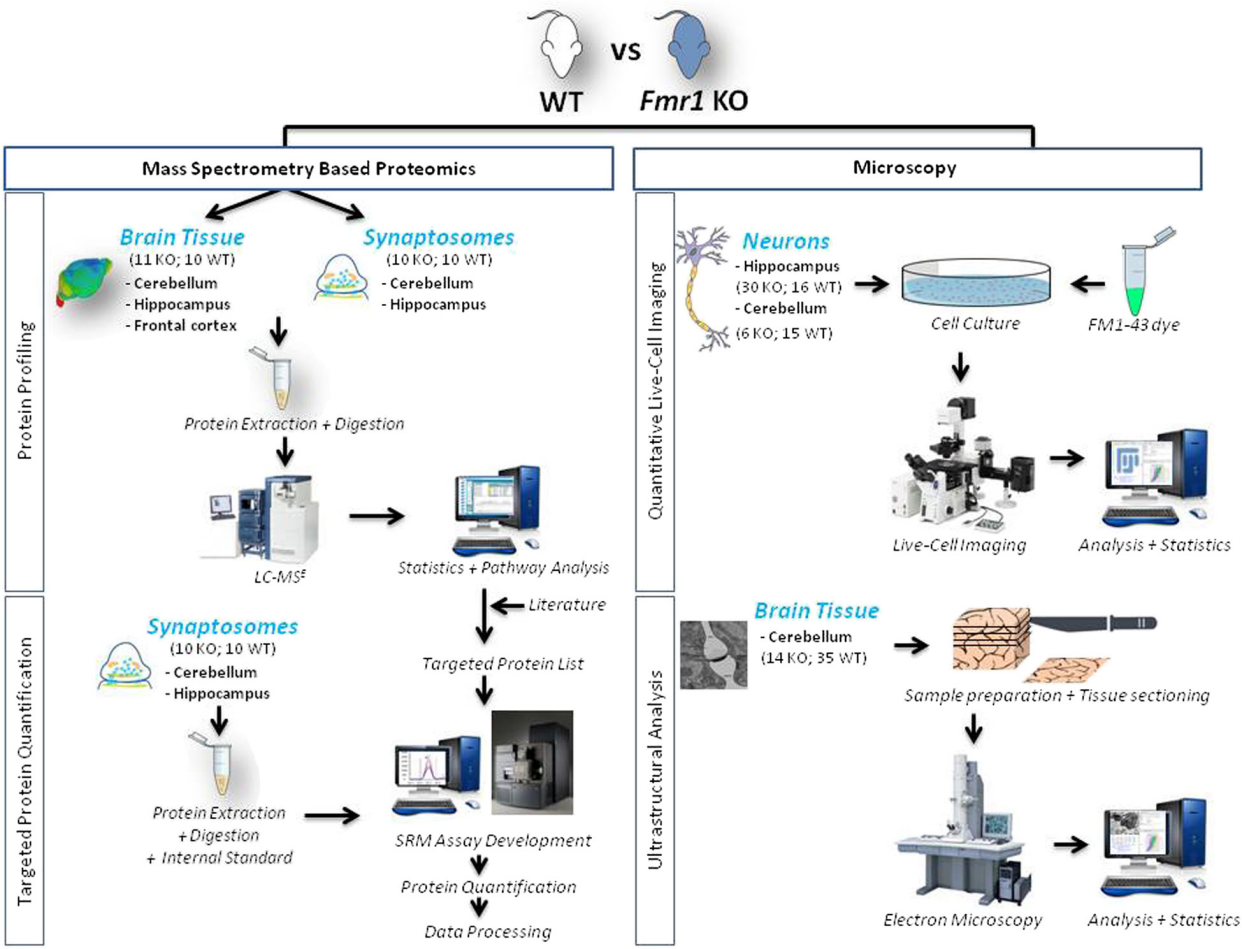
Overview of experimental design. In the mass spectrometry studies, the Fmr1 KO mouse model was compared with WT mice. Frontal cortex, hippocampal and cerebellar brain tissues were used for protein profiling using LC-MS^E^. For synaptosomes, both hippocampal and cerebellar tissues were used for LC-MS^E^ and SRM-MS studies. Data analysis resulted in the identification of significant protein changes. For live-cell imaging, neurons of the Fmr1 KO mouse model were cultured and compared with WT mice for both hippocampus and cerebellum. These neurons were stained with FM1-43 dye that specifically stains synaptic vesicles. For electron microscopy, Fmr1 was specifically knocked down in Purkinje cells and these cells were used for ultrastructural analysis. Data analysis was performed in instrument specific programmes, and statistical analysis was performed using R statistical programming language

In this study, a fragile X mental retardation 1 gene (*Fmr1*) KO mouse model was used to study synaptic changes in the hippocampus and cerebellum. In the first part, both brain tissue and synaptosomes were subjected to molecular profiling analyses using a combination of liquid-chromatography mass spectrometry (LC-MS^E^) and selected reaction monitoring mass spectrometry (SRM-MS) to detect and validate protein changes (Fig. 1). We found that in the absence of FMRP, most of the protein changes occurred at the synapse, which is in line with previous studies indicating differences in signal transduction, neuronal development and GABA/glutamate neurotransmission. Therefore, the second part of this study was focused on quantitative live-cell imaging investigating synaptic vesicle recycling in primary hippocampal and cerebellar neurons and electron microscopy to investigate potential presynaptic structure differences in the cerebellum. These studies indicated differences in the dynamics of synaptic vesicle turn-over, and together with the identified protein changes, we hypothesise that disturbances of synaptic dynamics are linked to fragile X syndrome.

## Methods

### Animal model

*Fmr1* knockout (KO) mice and wild type (WT) littermates on a congenic C57BL/6 background were provided by the Clinical Genetics group at Erasmus MC, The Netherlands. The mouse experiments were approved in advance by the Institutional Animal Welfare Committee (Erasmus MC, Rotterdam, The Netherlands). Mouse brain tissue samples used for LC-MS^E^ experiments were obtained by crossing FVB/Ant x heterozygous *Fmr1* KO(2) to test hybrid mice with 50 % FVB/Ant and 50 % C57BL/6 contributions [11]. Both lines were inbred (>10 times backcrossed). All mice were male, aged 14 weeks old, with an average weight of 30.2 +/− 2.0 g for *Fmr1* KO and 29.2 +/− 2.5 g for wild type (WT) mice and were tested on the ErasmusLadder (for data obtained with the ErasmusLadder see [12]). After the training period, 11 *Fmr1* KO and 12 WT male mice were killed and the bilateral hippocampi, frontal cortices and cerebella were dissected. All mice were sacrificed by cervical dislocation, and brains were dissected immediately.

*Fmr1* KO(2) mice and WT littermates [11] were used to obtain synaptosomal fractions as described by Schrimpf and colleagues [13]. Compared to the brain tissue samples, the background of these mice was C57BL/6 and backcrossing occurred for more than 10 generations. Synaptosomal fractions were obtained from the hippocampi (*n* = 10/group) and cerebella (*n* = 10/group), and these samples were used for homogenization and stepwise centrifugation to remove cell debris and to extract the synaptosomes [13].

For neuronal cultures, E18 *Fmr1* KO(2) and WT mouse embryos with a C57BL/6 background [11] were decapitated and both the hippocampi and the cerebella were dissected and used for neuronal cell culture (see Additional file 1, page 22). For electron microscopy, Purkinje cell-specific *Fmr1* KO mice with a C57BL/6 background were generated via deletion of the first coding exon of *Fmr1* through Cre-mediated recombination, as described by Koekkoek and colleagues [14].

### Protein digestion

Sample preparation was performed as previously described for both non-targeted and targeted studies (see Additional file 1, pages 1–4) [15, 16]. In short, samples were reduced and alkylated prior to tryptic digestion. Quality control (QC) samples were created by pooling all samples after alkylation.

### Label-free LC-MS^E^ protein profiling

Reverse-phase ultra performance-liquid chromatography (UPLC), using C-18 columns, was coupled to a Q-TOF Premier™ MS (Waters) system through a nano-electron spray ionization (ESI) online emitter (see Additional file 1, page 2). LC-MS^E^ data was processed using ProteinLynx Global Server v.2.5 (Waters Corporation) and Rosetta Elucidator v.3.3 (Rosetta Biosoftware, Seattle, WA). Criteria for protein identification were ≥3 fragment ions per peptide, ≥7 fragment ions per protein and ≥2 peptides per protein.

### Targeted protein quantification

Candidate proteins, chosen based on in silico pathway analyses of the LC-MS^E^ results, as described in Additional file 1, pages 1–15, as well as proteins associated with FMRP in the literature, were further validated and quantified using targeted selected reaction monitoring mass spectrometry (SRM-MS) on a Xevo TQ-S mass spectrometer coupled to a nanoAcquity UPLC system (Waters Corporation). Criteria for selecting tryptic peptides were based on peptide count, uniqueness and quality of transitions. Two peptides were selected for each target protein and isotopically labelled peptides were synthesised at JPT Peptide Technologies GmbH (Berlin, Germany). At least three transitions were measured for each peptide, and each sample was analysed in triplicate. Isotopically labelled synthetic peptides were spiked into the samples as an internal standard prior to MS analysis. The candidate proteins were validated and quantified with SRM mass spectrometry as described previously [15]. Due to the observation that most of the proteins identified in the initial LC-MS^E^ discovery study were located at the synapse, the proteomic profiles of synaptosome fraction were investigated using LC-MS^E^ as well as SRM for validation purposes.

### Statistical analysis of mass spectrometry data

Both LC-MS^E^ and SRM data were analysed in the R statistical programming language (version 3.1.1 [17]) using the MSstats package (Purdue University; West Lafayette, IN, USA), which provides wrapper functions to simplify the fitting of linear mixed effects models. The data of the identified proteins were log_2_ transformed and normalised for the intensities of the peaks. For the label-free LC-MS^E^ experiment, constant normalization was performed based on endogenous signals across runs among all proteins. Peptide transitions were excluded based upon a between-run-interference score <0.8, where the score was based on the correlation between mean of peptide by run and peptide transition intensity. Analysis was performed using linear mixed models to detect differentially expressed proteins between groups, as this approach can handle the hierarchical structure of the data. The interference for biological replicates and technical replicates was used in expanded scope, which expands the conclusion from the model to the population of biological units [18]. We considered results significant when proteins were found to be changed by more than 10 % and with a *p* value <0.05. These analyses resulted in *p* values, *q* values (Benjamini&Hochberg method) and ratio changes (*Fmr1*/WT).

For SRM-MS analysis, proteins with a peptide count (PC) >2, *p* value <0.05 and a ratio >10 % were included. Difference from the LC-MS^E^ analysis was that constant normalization was performed based on reference signals across runs among all proteins, and quantification was based on the ratio between endogenous and reference intensity. Data was analysed using a linear mixed model in restricted scope, which limits the conclusion from the model to observed biological units [18].

Furthermore, protein-protein interaction networks were constructed from the significantly changed proteins by finding first-degree interacting neighbours as described in the Additional file 1, pages 16–21.

### Analysis of live-cell imaging and electron microscopy

The synaptic boutons, visualized in primary neurons as described in the Additional file 1, pages 22–25, were analysed by identifying regions of interest (ROIs) using Fiji [19]. Sample movement was corrected using StackReg [20], and synaptic boutons were detected and tracked using a semi-automated procedure implemented in Fiji plug-in (available from http://smal.ws/wp/software/sosplugin/[21]). Briefly, synaptic boutons were detected by fitting Gaussian distributions with sigma corresponding to bouton sizes ranging from 300–800 nm. To differentiate between synaptic boutons and trafficking synaptic vesicles, tracks were created using a nearest-neighbour linking approach for objects moving less than 500 nm within three frames. Tracks were visualized and quantified with the MTrackJ plug-in of Fiji [22]. The identification of different unloading profiles, the weak and strong unloading synaptic boutons, was performed as described before [23].

In R statistical programming language (version 3.1.1 [17]), the MTrackJ results and background values were imported and relevant tracks were selected based on starting time point and minimum time point length. Baseline correction was performed by normalizing the tracks for baseline intensity. To identify the two different unloading profiles, tracks were hierarchically clustered in sub-populations based on the decrease of signal immediately after stimulation. The threshold criteria for clustering were (1) a decrease of the slope after stimulation of 25 %, (2) a relative amount of variation based on a ratio stdev/mean intensity lower than 0.5 and (3) stability of the baseline slope. Statistical analysis was performed using a *t* test with permutations to look for differences in the amount of synaptic boutons and analysis of variance (ANOVA) to compare the regression lines of the synaptic bouton dye release.

Furthermore, the ultrastructural data obtained by electron microscopy (Additional file 1, page 26) were statistical analysed in R using a *t* test with permutations (AZ length, presynaptic area and vesicle density) and correlation (density with AZ length).

## Results

### Quantitative LC-MS^E^ proteomic profiling of mouse frontal cortex, hippocampus and cerebellum

LC-MS^E^ profiling was carried out to determine the effects of the absence of the FMRP protein in mouse brain. Previous studies have indicated that the cerebellum [14, 24] and hippocampus [7] are prominently affected in FXS. Moreover, since frontal cortex dysfunction is known to play a key part in autism [25], we investigated the frontal cortex additionally. After data filtering, the analysis resulted in the identification of 553 (frontal cortex), 705 (hippocampus) and 536 (cerebellum) proteins, respectively. Following data quality assessment, 24 proteins were significantly different in the frontal cortex, 14 in the hippocampus and 11 in the cerebellum (Additional file 1, page 6). GO-enrichment analysis was performed to detect first-degree neighbour proteins using all significantly changed proteins with a ratio *Fmr1* KO/WT of >10 % (Additional file 1, page 16). The significant findings of the profiling study were predominantly proteins located at the synapse, such as ATPases and proteins important for neurotransmitter signalling (Additional file 1, page 9).

### Quantitative LC-MS^E^ proteomic profiling of synaptosome fractions isolated from the hippocampus and cerebellum

In the synaptosome fraction study, the hippocampus and cerebellum were prioritised as these are predominantly affected in FXS. LC-MS^E^ analysis of mouse synaptosomes resulted in the identification of 1114 (hippocampus) and 1040 (cerebellum) proteins, respectively. After data filtering, the number of significant proteins were 23 (hippocampus) and 13 (cerebellum) (Additional file 1, page 11). In GO enrichment, fewer networks are indicated for the synaptosome fractions compared to brain tissue, as fewer significant proteins were indicated (Additional file 1, page 20). The main protein changes are involved in synaptic signalling, neurotransmission, synaptic vesicles and neuron development (Additional file 1, page 14). Overlap of significantly altered networks between brain tissue and synaptosome fractions indicated protein dephosphorylation and synaptic signalling, such as glutamate pathways and dendrite morphogenesis, as changed in common (Additional file 1, page 21). Therefore, proteins involved in synaptic signalling as found in the protein profiling study of the synaptosome fractions and in brain tissue (Additional file 1, page 9, 14) were taken forward for targeted SRM analysis.

### Targeted SRM-MS validation of hippocampal and cerebellar synaptosome fractions

An SRM-MS assay panel was developed to validate changes related to signal transduction, neuronal development and neurotransmission, including isoforms of the theme proteins and proteins known to relate to FXS from the literature. The results show that most of the significantly changed proteins in the hippocampus were decreased in abundance, while proteins significantly changed in the cerebellum were increased (Table 1). In the hippocampus, a significant decrease was found for proteins associated with neuronal development and GABA/glutamate neurotransmission, as well as significantly different levels of two ATPases. Most significant protein changes were observed in the cerebellum, with a general increase of abundance in ATPases and GABA/glutamate-related proteins. Furthermore, several neuronal development proteins were significantly different. FMRP-related protein CYFP1 was found to be significantly changed in abundance in both the hippocampus and cerebellum, while CYFP2 and FXR2 were only changed in the cerebellum (Table 1).

**Table 1:** Protein changes validated using SRM-MS in the mouse brain synaptosome fractions

	HC			CB		
Protein name	Ratio Fmr1 KO/WT	*p* value	*q* value	Ratio Fmr1 KO/WT	*p* value	*q* value
Signal transduction						
Plasma membrane calcium-transporting ATPase 2 (AT2B2)^a, b, e^	1.11	0.6403	0.8270	1.16	0.2314	0.2953
Sodium potassium transporting ATPase alpha 1 (AT1A1)^a^	0.99	0.8710	0.9194	1.32	2.60*10 ^*−11*^	3.20*10 ^*−10*^
Sodium potassium transporting ATPase alpha 3 (AT1A3)^a^	0.74	*0.0076*	*0.0480*	1.66	2.50*10 ^*−07*^	1.03*10 ^*−06*^
Sodium potassium transporting ATPase beta 1 (AT1B1)^a^	1.01	0.9524	0.9524	1.27	2.63*10 ^*−10*^	2.43*10 ^*−09*^
Sodium potassium transporting ATPase beta 2 (AT1B2)^a^	1.21	*0.0509*	0.1874	1.19	*0.0122*	*0.0215*
Synaptic vesicle glycoprotein 2A (SV2A)^b, d^	1.02	0.6529	0.8270	1.20	*0.0002*	*0.0005*
Synaptic vesicle glycoprotein 2B (SV2B)^b^	0.86	0.1754	0.3715	0.92	0.5270	0.5909
Neuronal development						
Brain acid soluble protein 1 (BASP1)^c^	0.77	<2.60*10 ^*−11*^	<2.60*10 ^*−11*^	1.75	8.15*10 ^*−07*^	3.02*10 ^*−06*^
Neuromodulin (NEUM)^a^	0.88	*0.0751*	0.1874	0.77	*0.0071*	*0.0146*
Neurotransmission: GABA/glutamate-related proteins						
BTB POZ domain containing protein KCTD12 (KCD12)^a^	0.73	0.38*10 ^*−05*^	0.0004	1.17	*0.0098*	*0.0192*
Glutamate receptor 1 (GRIA1)^b, d^	0.83	*0.0789*	0.1874	1.77	1.35*10 ^*−07*^	6.24*10 ^*−07*^
Glutamate receptor 3 (GRIA3)^b, d^	0.87	0.1760	0.3715	1.34	*0.0160*	*0.0269*
Succinate semialdehyde dehydrogenase (SSDH)^a^	1.06	0.3880	0.5897	0.93	0.3833	0.4432
Vesicular glutamate transporter 1 (VGLU1)^a^	1.02	0.8017	0.8934	1.23	*0.0222*	*0.0342*
Excitatory amino acid transporter 1 (EAA1)^a^	1.14	0.3178	0.5251	1.13	0.3431	0.4095
Glutamate decarboxylase 1 (DCE1)^b^	0.94	0.6107	0.8270	0.95	0.7279	0.7782
Glutamate decarboxylase 2 (DCE2)^b, d^	0.81	0.3422	0.5419	1.27	0.2416	0.2979
FMRP-related proteins						
Cytoplasmic FMR1-interacting protein 1 (CYFP1)^b, d^	0.65	*0.0205*	0.0973	1.45	*0.0437*	*0.0621*
Cytoplasmic FMR1-interacting protein 2 (CYFP2)^b^	1.04	0.7761	0.8934	1.41	*0.0217*	*0.0342*
Fragile X mental retardation syndrome-related 2 (FXR2)^c^	1.04	0.7108	0.8713	1.26	*0.0070*	*0.0146*

* this is the multiply sign

The table includes Uniprot ID, ratios (calculated based on average), *P* values and adjusted *P* values (Q). Significant proteins are indicated in italics

^a^Proteins indicated in LC-MS^E^ brain study

^b^Proteins indicated in LC-MS^E^ synaptosome study

^c^Proteins associated with FMRP in literature

^d^Isoforms of indicated proteins in B or S

^e^Family member of proteins indicated in B or S

### Identification of synaptic boutons using live-cell imaging

We could distinguish different unloading frequencies of the synaptic boutons, which resulted in strong (35 % intensity decrease after stimulation compared to baseline) and weak (18 % intensity decrease) FM1-43 unloading profiles as described in Bartolome-Martin [23] (Additional file 1, pages 22–24). After identification of the different unloading profiles, the number of synaptic boutons was counted, which showed no differences in the number of synaptic bouton unloading profiles in either the hippocampus or the cerebellum (Additional file 1, page 25). This result suggests that the number of synaptic boutons in cultured neurons are comparable between the *Fmr1* KO and WT mice, and as dye loading and unloading involve the same stimulation protocol (50 mM K^+^, 5 min), any differences in unloading kinetics are likely to reflect an alteration in synaptic vesicle dynamics.

### Synaptic bouton dynamics in live-cell imaging

**Fig. 2:**
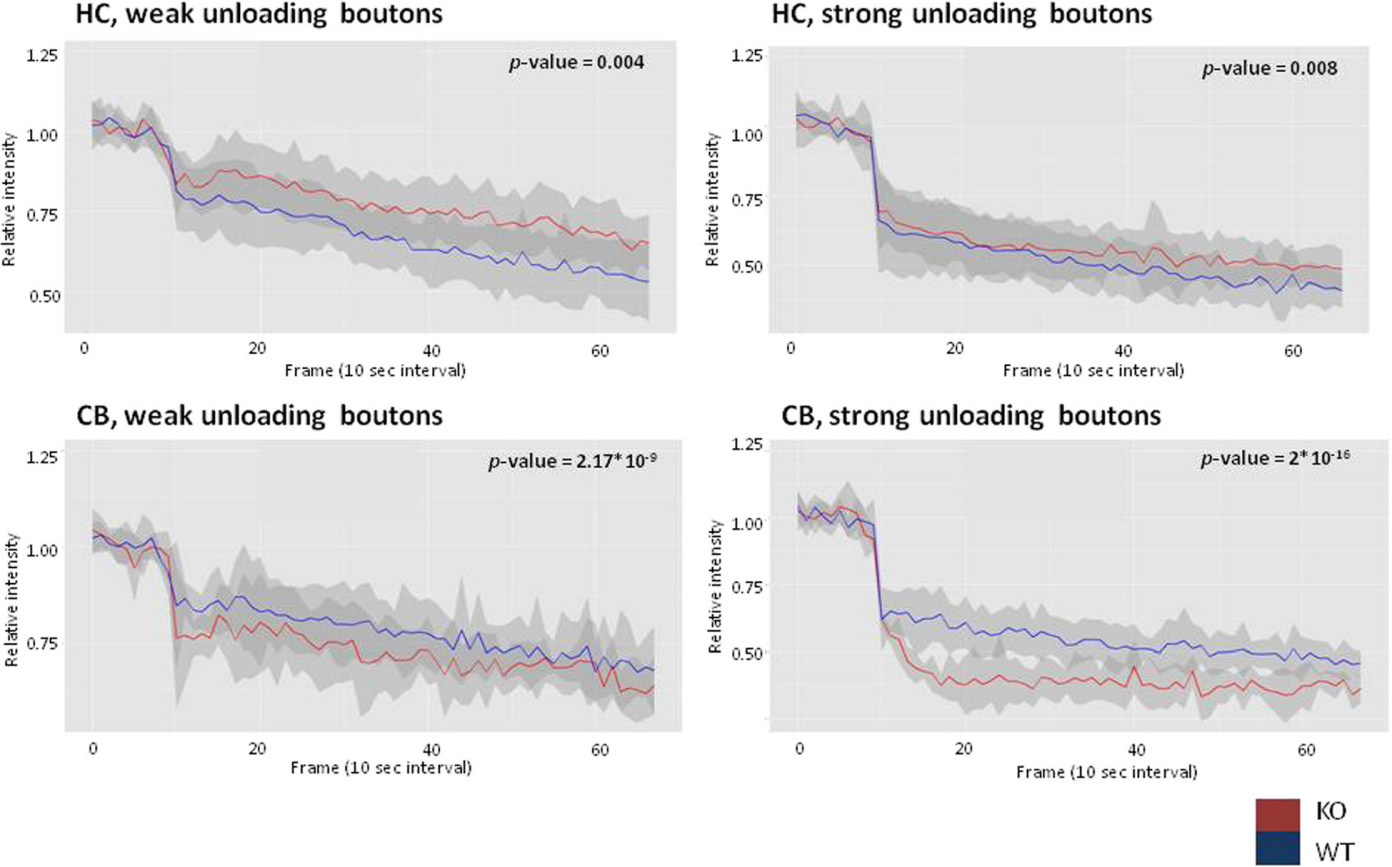
Differences in synaptic bouton unloading of FM1-43 dye in hippocampal (HC) and cerebellar (CB) neurons. (*top left*) Weak unloading boutons are less synaptically active, which translates into the release of fewer synaptic vesicles and neurotransmitters. Strong unloading boutons have a greater number of synaptic vesicle exocytosis and are therefore expected to release neurotransmitters more actively. Kinetics of FM1-43 unloading of weak unloading boutons in the hippocampus showed a significant difference with a *p* value of 0.004 during stimulation with 50 mM KCl and (*top right*) a significant difference with a *p* value of 0.008 for strong unloading boutons in the hippocampus. In the cerebellum, the weak unloading boutons showed a highly significant change with a *p* value of 2.17 × 10^−9^ (*below left*), as well as a highly significant change with a *p* value of 2 × 10^−16^ for strong unloading of FM1-43 dye from synaptic boutons (*below right*). The *p* values were obtained by regression analysis with ANOVA comparing Fmr1 KO with WT mouse primary neurons

In live-cell imaging, the weak unloading boutons had an intercept of 0.91 relative intensity (RI) (*Fmr1* KO) and 0.79 RI (WT) in the hippocampus and 0.81 RI (*Fmr1* KO) and 0.88 RI (WT) in the cerebellum. The intercepts of the strong unloading boutons were 0.64 RI (*Fmr1* KO) and 0.61 RI (WT) for hippocampal neurons and 0.41 RI (*Fmr1* KO) and 0.59 RI (WT) for cerebellar neurons. Determining the difference in rate of dye release showed a significant decrease of both unloading profiles in *Fmr1* KO compared to WT mice in the hippocampus (weak unloading boutons: *p* value = 0.004; strong unloading boutons: *p* value = 0.008; Fig. 2). The synaptic vesicle dynamics in the cerebellum showed a significant increase of unloading activity in *Fmr1* KO mice compared to WT (weak unloading boutons: *p* value = 2.17 × 10^−9^; strong unloading boutons: *p* value = 2 × 10^−16^; Fig. 2). This implies that *Fmr1* KO cerebellar neurons have a higher turn-over rate of synaptic vesicle recycling.

### Ultrastructural analysis of cerebellar Purkinje cells

**Fig. 3:**
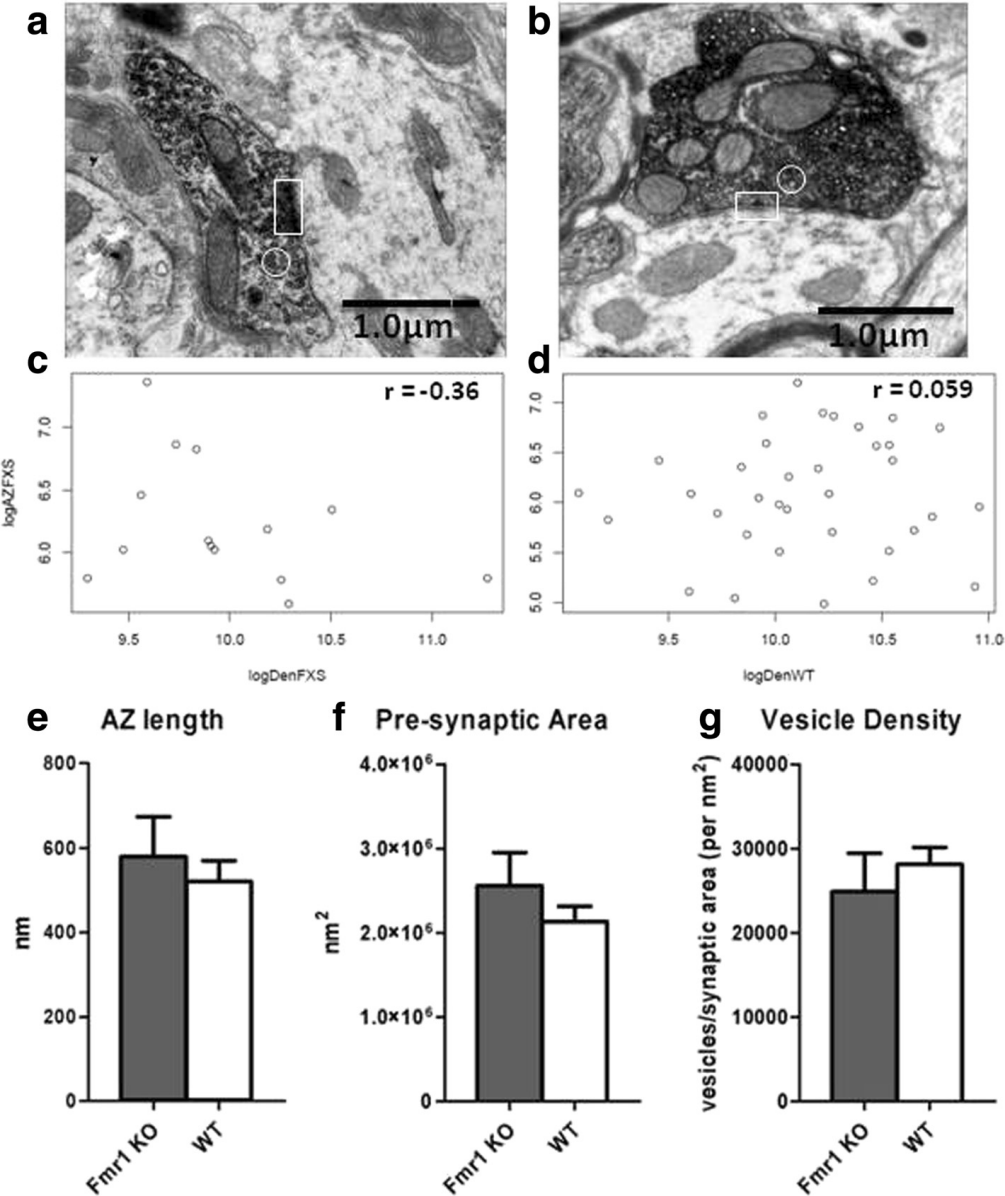
Ultrastructural analysis of Purkinje cell synapses. Fmr1 KO (**a**) as compared to WT (**b**) Purkinje cell synapses revealed a negative correlation of density vs. active zone (AZ) length in Fmr1 KO (**c**), while this was positive for WT (**d**). **e**–**g** No significant differences were observed for AZ length, presynaptic area and vesicle density. In **a** and **b**: *white rectangle*= AZ; *white circle*= synaptic vesicles; and *dark stained area*= presynaptic area

Statistical *t* test with permutation of the labelled Purkinje cell terminals (Fig. 3) was performed and showed no significant difference for active zone (AZ) length (*p* value = 0.583), presynaptic area (*p* value = 0.357) and synaptic vesicle density (*p* value = 0.543). When synaptic vesicle density was correlated with the AZ length, we found a negative correlation of *r* = −0.36 (*p* value = 0.206) for the *Fmr1* KO and a correlation of *r* = 0.059 (*p* value = 0.7364) for WT mice. These results imply that the unloading activity at the synapse as observed in live-cell imaging is caused by a higher turn-over rate of synaptic vesicle recycling and not by an increased number of synaptic vesicles.

## Discussion

Here, we present a comprehensive study combining proteomic and microscopic investigations in an *Fmr1* KO mouse model. We employed orthogonal quantitative and qualitative proteomic approaches, live-cell visualization of synaptic vesicle dynamics and ultrastructural analyses of synapses to investigate protein alterations and their functional effects at the synapse in the context of FMRP deletion. Our findings provide evidence that molecular processes in synaptic transmission are affected in the hippocampus and cerebellum of the *Fmr1* KO mouse model. Surprisingly, most of the significantly affected proteins were identified in the cerebellum, which comprises changes in proteins associated with signal transduction, neuronal development and GABA/glutamate neurotransmission. The finding that the cerebellum is a prominently affected brain region is in line with behavioural findings using the ErasmusLadder, demonstrating major deficits in associative motor learning in *Fmr1* KO mice [12]. In the microscopy studies, we found that a lack of FMRP leads to changes in synaptic vesicle unloading dynamics, with a strong increase of synaptic vesicle turn-over in cerebellar neurons and a decrease in hippocampal neurons. Therefore, alterations in synaptic vesicle dynamics due to loss of FMRP likely contribute to aberrant synaptic transmission in FXS patients. Recently, Myrick and colleagues found protein synthesis-independent presynaptic function for FMRP, using *Drosophila melanogaster* models with a missense mutation of a patient with a partial FXS phenotype [26]. Our results together with previous studies suggest that FMRP regulates translation of proteins important for synaptic plasticity, indicating that FMRP has specific pre-and postsynaptic functions that contribute to different components of the FXS pathophysiology [27].

The proteomic study showed that FMRP loss resulted in changes in protein levels at the synapse, specifically in proteins involved in signal transduction, neuronal development and neurotransmission. The SRM-MS study of the synaptosome fractions indicated that proteins associated with signal transduction mainly relate to ATPases, which are proteins that contribute to creating an ion gradient across the synaptic membrane and therefore are important for synaptic transmission [28]. A functional link between glutamate action and ATPase activity can be inferred from the observation that glutamate, through metabotropic receptors, is able to increase ATPase activity in Purkinje neurons [29]. In the cerebellum, both the glutamate and GABA receptors were increased in expression, whereas they were decreased in the hippocampus, which might implicate that the excitability of neurons is changed. Changes in glutamate and GABA have been observed before in fragile X animal investigations, resulting in the mGluR theory and hypo-inhibition of GABA [7]. Moreover, VGLU1, a protein that mediates the uptake of glutamate into synaptic vesicles, and SV2A, important for fusion of synaptic vesicles to the membrane, were significantly increased in the cerebellum. A previous proteomic study using primary *Fmr1* KO hippocampal mouse neurons also identified changes in the SV2A protein [30]. Other synaptic vesicle changes in the hippocampus indicate smaller active zones and pools of clustered vesicles [31], a decrease of vesicle recruitment [8] and abnormal short-term plasticity [10]. In addition, synaptic plasticity regulators BASP1 and NEUM, which are important for synaptic vesicle recycling, were found to be significantly changed in both the hippocampus and cerebellum, of which BASP1 is a consistent finding observed in previous studies in the hippocampus [31]. Overall, this proteomic investigation provides evidence that loss of FMRP in the mouse brain alters synaptic signalling due to changes the in regulation of membrane potential and neurotransmitter release, which is in line with previous findings [26]. Using SRM-MS, we also found a significant change in the expression of FMRP-associated protein CYFP1 [32], both in hippocampal and cerebellar synaptosome fractions. Changes in FXR2 [33] correlated with changes in CYFP2 [32], which could be due to their interaction [34].

Investigating synaptic transmission using microscopy showed an increase of synaptic vesicle recycling in cerebellar neurons, which is held true for both boutons with weak and strong unloading. The majority of cerebellar neurons consist of Purkinje cells, which represent the main GABAergic inhibitory neuronal output from the cerebellum. An increase of synaptic vesicle recycling in cerebellar neurons might represent an increase of inhibitory output from the cerebellum. Since the amount of synaptic vesicles, active zone length and presynaptic area were not changed in the Purkinje cell axon terminals, the changes in synaptic vesicle recycling is mainly dynamic. These findings raise the question to what extent the enhanced LTD observed in *Fmr1* KO Purkinje cells [14] reflects a compensatory process rather than a primary defect. Indeed, it is possible that homeostatic mechanisms through the olivocerebellar loop regulate the level of synaptic plasticity in Purkinje cells so as to stabilise the output of the cerebellar nuclei [35].

Decreased synaptic transmission in mouse amygdalae, neocortex and rat dorsal root ganglia have been observed before [36–38]. These studies together with the increase in long-term depression (LTD), as stated by the mGluR theory [7], imply a decrease of synaptic transmission in the FXS hippocampus. Interestingly, the study of Deng (2011) suggested increased synaptic transmission in the hippocampus, due to increased short-term plasticity (STP) [10]. However, Klemmer (2011) found reduced synaptic transduction resulting from a decreased number of synaptic vesicles, a shorter active zone length and a decrease of STP [31]. Recently, the study of Telias et al. indicated fewer synaptic vesicles and spontaneous synaptic activity in FXS human embryonic stem cells [39]. In our study, we showed that FMRP depletion causes a decrease of synaptic vesicle recycling in both strong and weak unloading boutons in the hippocampus, while the amount of synaptic boutons remained unchanged between *Fmr1* KO and WT mice.

Only male mice were investigated in this study. Therefore, the proteomic changes relevant to ASD highlight the changes seen in the male sex. Although only *Fmr1* KO mice were investigated, differences in mouse genetic background between the tissue and synaptosome samples (see “Methods” section) might influence the results.

## Conclusions

**Fig. 4:**
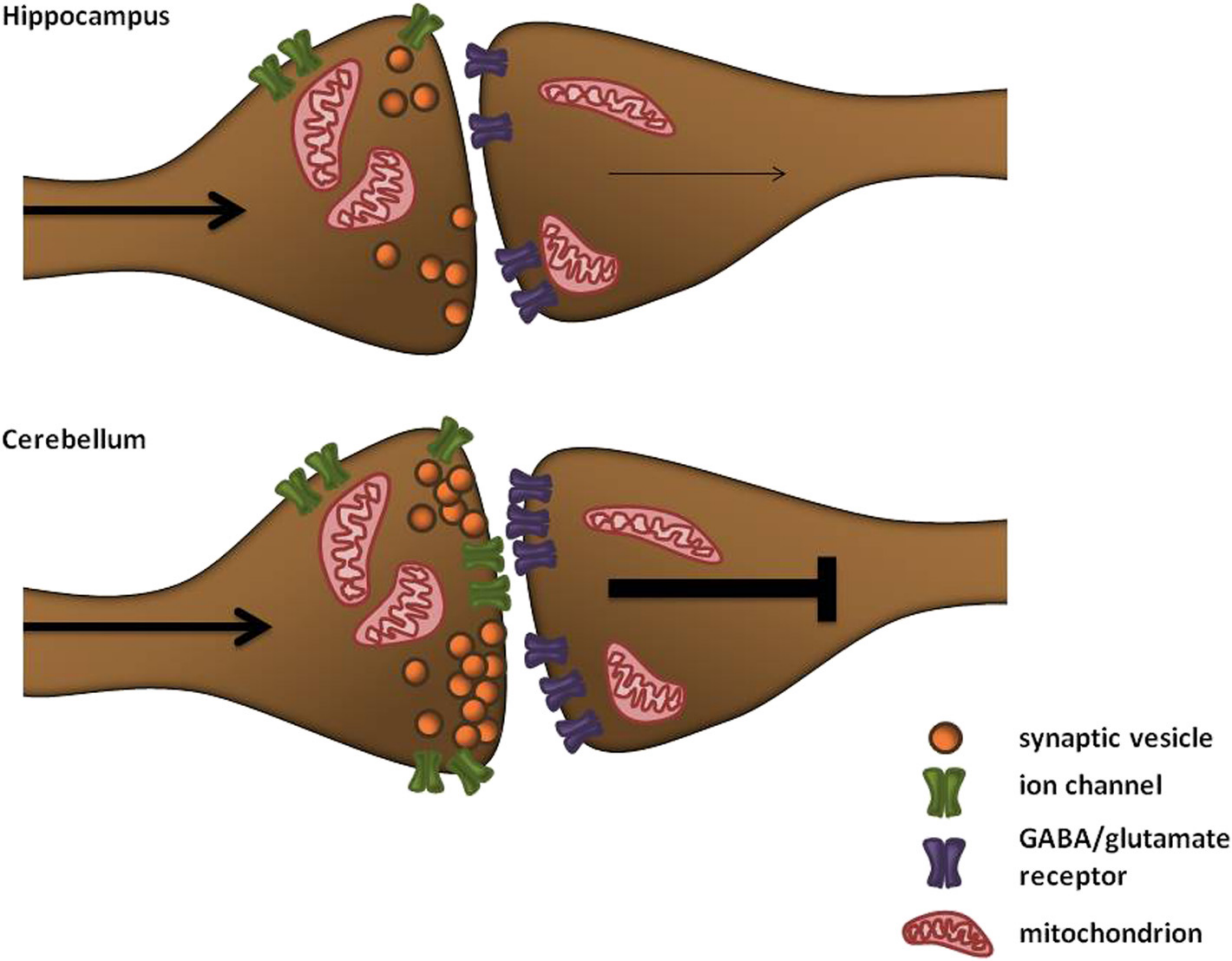
Changes observed in hippocampal and cerebellar synapses using mass spectrometry and microscopy studies. In the hippocampus (*above*), significant changes were observed for ATPases and a significant decrease of GABA receptor proteins. Furthermore, the microscopy studies indicated decreased synaptic vesicle release in hippocampal neurons. This might indicate decreased excitatory synaptic transmission in the hippocampus. A significant increase of ATPases, GABA and glutamate receptors and synaptic vesicle proteins were observed in cerebellar synapses (*below*). This increase in synaptic activity was also supported by the microscopy studies, in which cerebellar neurons show an increase of synaptic vesicle dynamics, suggesting an increased inhibitory output from the cerebellum

Taken together, our findings demonstrate that a lack of FMRP is associated with differences in synaptic vesicle dynamics in both hippocampal and cerebellar neurons (Fig. 4). Synaptic vesicles are critical for synaptic transmission in the nervous system, and inefficient synaptic transmission in the hippocampus and cerebellum could impair memory formation and motor learning in fragile X and possibly autism spectrum disorder (ASD) patients.

## Additional file

Additional file 1: Supplementary methods information, figures of principal component analysis (PCA), GO term analysis heatmap, live-cell imaging setup and number of synaptic boutons, and tables with a full list of significant changed proteins in mouse brain tissue and synaptosome fractions in LC-MS^E^ and GO term enrichment results. (DOCX 405 kb)

## Abbreviations

AT1A1: sodium potassium transporting ATPase alpha 1
AT1A3: sodium potassium transporting ATPase alpha 3
AT1B1: sodium potassium transporting ATPase beta 1
AT1B2: sodium potassium transporting ATPase beta 2
AT2B2: plasma membrane calcium-transporting ATPase 2
AZ: active zone
BASP1: brain acid soluble protein 1
CB: cerebellum
CYFP: cytoplasmic FMRP-interacting protein
DCE1: glutamate decarboxylase 1
DCE2: glutamate decarboxylase 2
EAA1: excitatory amino acid transporter 1
FM1-43: n-(3-triethylammoniumpropyl)-4-(4-(dibutylamino) styryl) pyridinium dibromide
*Fmr1*: fragile X mental retardation 1 gene
FMRP: fragile X mental retardation protein
FXR2: fragile x mental retardation syndrome-related 2
FXS: fragile X syndrome
GABA: gamma-aminobutyric acid
GRIA: glutamate receptor
H1T: histone H1t
HC: hippocampus
KCD12: BTB POZ domain- containing protein KCTD12
KO: knockout
LC-MS^E^: liquid-chromatography mass spectrometry
mGluR: metabotropic glutamate receptor
NEUM: neuromodulin
PC: peptide count
Q-TOF: quadrupole time-of-flight
RI: relative intensity
ROIs: regions of interest
SRM-MS: selected reaction monitoring mass spectrometry
SSDH: succinate semialdehyde dehydrogenase
STP: short-term plasticity
SV2A: synaptic vesicle glycoprotein 2A
SV2B: synaptic vesicle glycoprotein 2B
VGLU1: vesicular glutamate transporter 1
WT: wild type
