# Supplementary material for: Synaptic vesicle dynamic changes in a model of fragile X
Source: Mol Autism. 2016 Mar 1;7:17. doi: 10.1186/s13229-016-0080-1 (PMC4772588; doi:10.1186/s13229-016-0080-1)
Supplement: Additional file 1: — Supplementary methods information, figures of principal component analysis (PCA), GO term analysis heatmap, live-cell imaging setup and number of synaptic boutons, and tables with a full list of significant changed proteins in mouse brain tissue and synaptosome fractions in LC-MSE and GO term enrichment results. (DOCX 405 kb) [file 13229_2016_80_MOESM1_ESM.docx]

Appendix: sYNAPTIC VESICLE DYNAMIC CHANGES IN A MODEL OF FRAGILE x

# Supplementary Information

## Label-free LC-MS^E^ profiling

Label-free LC-MS^E^ profiling was performed for the indication of protein level differences in brain tissue and synaptosomal fraction of a *Fmr1* KO mouse model. In the experiment, all reagents were obtained from Sigma-Aldrich (Poole, UK) unless specified otherwise, and sample preparation and LC-MS^E^ analysis were performed as previously described [1, 2]. Protein extraction was performed by addition of fractionation buffer (7M urea, 2M thiourea, 4% 3-[(3-cholamidopropyl)dimethylammonio]-1-propanesulfonate, 2% ASB14 and 70 mM dithiotreitol (DTT)), followed by sonication for 10 seconds using a Branson Sonifier 150 (Thistle Scientific; Glasgow, UK) and vortexing for 30 minutes at 4°C. The homogenates were centrifuged for 3 minutes at 17,000g and the supernatants collected for precipitation of the proteins using 4:1 volumes ice-cold acetone. The resulting pellets were suspended in 100 μL of 50 mM NH_4_HCO_3_ (pH 8.0). Disulfide groups on proteins were reduced into sulfhydryl groups in the expense of S-S bridges by incubation with 100 mM DTT for 30 minutes at 60°C and alkylated with 200mM iodacetamide for 30 minutes at 37°C. Quality control (QC) samples were created by pooling all samples for use, and sample preparation of all samples and QCs proceeded with tryptic digestion of the proteins. Proteins were cleaved into peptides by incubation with 1:50 (trypsin:protein) porcine trypsin (Promega; Madison, WI, USA) for 17 hours at 37°C and stopped after 16 hours by addition of 0.80μL of 8.8M HCl. Samples were stored at -80°C. Prior to mass spectrometry analyses, 0.1% formic acid was added to a final concentration of 0.12 μg/μL protein.

Peptides were separated by reversed-phase ultra performance-liquid-chromatography (UPLC), which was accomplished using the splitless Waters’ nanoACQUITY UPLC system (10kpsi). For chromatography, all solvents were mass spectrometry grade (Fisher Scientific) and C-18 columns were used during the experiment. The nanoUPLC system was coupled to a MS system through a nano electron spray ionization (ESI) online emitter (7 cm length, 10μm tip; NewObjective; Woburn, MA), which causes the ionization of peptides before they can be analysed by a Q-TOF PremierTM MS (Waters). The Q-TOF MS was operated in positive ion nanoES V mode with alternate scanning data independent acquisition (MSE) at a mass resolution of 10,000. During the LC-MS^E^ process, LockSpray was used with reference compound Glu-fibrinopeptide B (Sigma) to correct for any drift in mass calibration.

LC-MS^E^ data was processed using the ProteinLynx Global Server v.2.5 (Waters Corporation) and Rosetta Elucidator v.3.3 (Rosetta Biosoftware, Seattle, WA) for time and mass/charge alignment of mass spectrometer data as described previously [3]. The *Mus musculus* complete proteome fasta sequence Integr8 database was used for the assignment of protein identities. Quantitative peptide measurements for each replicate were normalized against the total ion volume of all deconvoluted spectra. The criteria for protein identification were set to ≥ 3 fragment ions per peptide, and ≥ 7 fragment ions per protein and ≥ 2 peptides per protein. The data were also searched against a randomised decoy database, which was created using the original database, thus conserving amino acid frequencies. Only peptides that were present in all samples of each treatment group were considered for further analysis. The final part of the data processing was a principal component analysis (PCA; SIMCA P+ version 2.12; Umetrics; Malmö, Sweden), which was used to identify unwanted variability due to sample non-homogeneity or inconsistent manipulation during the preparation and analytical stages (**supplementary figure S1a and S1b**). Testing for outlying samples resulted in removal of one sample from both the mouse brain hippocampus and cerebellum, and one sample from the synaptosome hippocampus.

After data filtering, the analysis resulted in the identification of 553 (frontal cortex), 705 (hippocampus) and 536 (cerebellum) proteins in mouse brain tissue, and 1114 (hippocampus) and 1040 (cerebellum) proteins in mouse synaptosome fractions. In brain tissue, following data quality assessment with FC>10%, 24 proteins were significantly different in the frontal cortex, 14 in the hippocampus, and 11 in the cerebellum (**supplementary table S1**). These significant findings of the brain tissue were predominantly proteins located at the synapse, such as ATPases and proteins important for neurotransmitter signalling (**supplementary table S2**). Further protein changes were associated with metabolism and cell connectivity. Due to the initial results in the brain tissue profiling study, a synaptosome fraction study was performed to investigate proteomic profiles using both LC-MS^E^ and SRM. In this supplementary part, only the LC-MS^E^ study of the synaptosome fractions are shown, as the SRM study is discussed in the paper. In the synaptosome fraction study, the hippocampus and cerebellum were prioritized as these are affected in FXS. The same LC-MS^E^ procedure for the synaptosome fractions resulted in 23


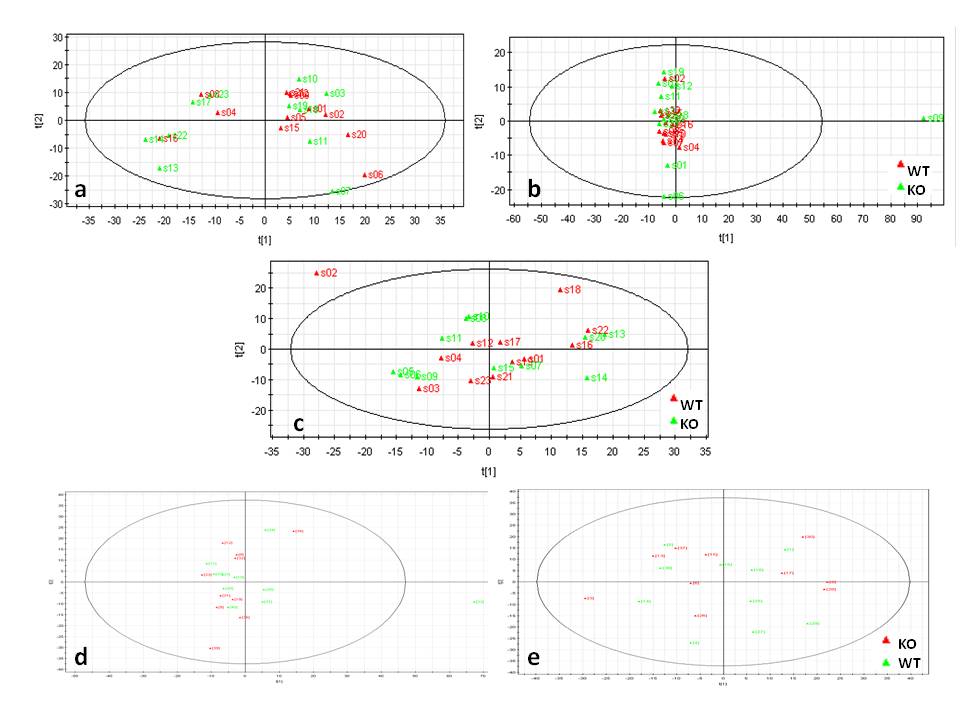
(hippocampus) and 13 (cerebellum) proteins found to be altered by more than 10% were (**supplementary table S3**). The main protein changes in the synaptosome fractions are involved in synaptic signalling, neurotransmission, synaptic vesicles and neuron development (**supplementary table S4**).

**Supplementary figure S1a. Principal component analysis (PCA) of brain tissue**. Each point represents one sample. In mouse frontal cortex (a), no outliers were detected. Hippocampus (b) and cerebellum (c) showed one sample that lay outside the 95 per cent confidence interval (ellipse)(hippocampus: s09; cerebellum: s02). Outliers were excluded from further analysis.


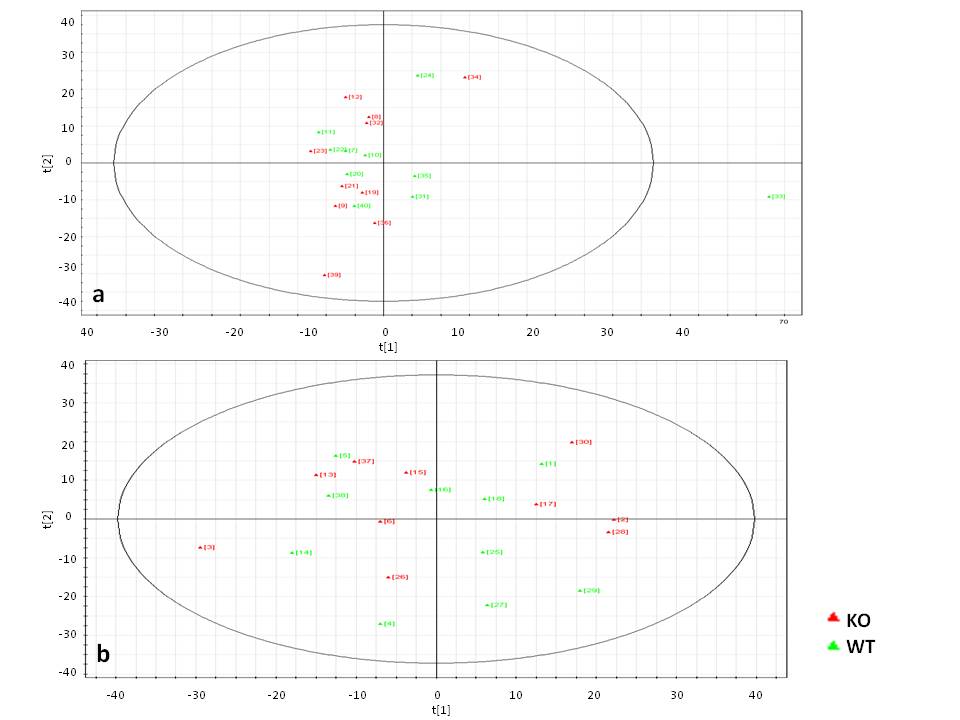


**Supplementary figure S1b. Principal component analysis (PCA) of synaptosome fractions**. Each point represents one sample. In the mouse synaptosomes, one outlier was detected in the hippocampus (d) (s33) and none in the cerebellum (e). Outliers were excluded from further analysis.

| **Supplementary table S1. Analysis of protein levels in mouse brain tissue of Fmr1 KO and WT** | | | | |
| --- | --- | --- | --- | --- |
| **Protein name** | **No. of peptides** | **ratio *Fmr1* KO/WT** | ***p*-value** | **biological function** |
| ***Frontal cortex*** |  |  |  |  |
| Creatine kinase B (KCRB) | 31 | 0.90 | 0.0012 | creatine kinase |
| Sarcoplasmic endoplasmic reticulum calcium ATPase 2 (AT2A2) | 4 | 0.84 | 0.0059 | ATPase |
| L-lactate dehydrogenase B chain (LDHB) | 32 | 0.92 | 0.0063 | glycolysis |
| Tubulin beta-2A chain (TBB2A) | 16 | 0.93 | 0.0080 | tubulin |
| Sodium/potassium-transporting ATPase subunit beta-1 (AT1B1) | 20 | 0.93 | 0.0102 | ATPase |
| Fructose-bisphosphate aldolase A (ALDOA) | 29 | 0.92 | 0.0103 | glycolysis |
| Ubiquitin 60S ribosomal L40 (RL40) | 13 | 0.87 | 0.0104 | translation |
| Lysine-specific histone demethylase 1A (KDM1A) | 2 | 0.86 | 0.0109 | transcription |
| Cytochrome c oxidase 5A (COX5A) | 5 | 0.73 | 0.0112 | mitochondrion |
| Ras related Rab 43 (RAB43) | 10 | 0.82 | 0.0115 | transport |
| Complex I intermediate-associated protein 30 (CIA30) | 4 | 0.82 | 0.0115 | mitochondrion |
| Tubulin alpha-4A chain (TBA4A) | 19 | 0.92 | 0.0123 | Tubulin |
| Calcium calmodulin dependent kinase II delta (KCC2D) | 8 | 0.87 | 0.0128 | calcium regulation |
| Succinate semialdehyde dehydrogenase (SSDH) | 2 | 1.16 | 0.0135 | neurotransmitter |
| Myelin basic protein (MBP) | 24 | 0.92 | 0.0163 | Myelin |
| Hippocalcin like 4 (HPCL4) | 3 | 0.74 | 0.0182 | rhodopsin |
| Cofilin 2 (COF2) | 6 | 0.75 | 0.0193 | cytoskeleton |
| Serine threonine kinase 4 (STK4) | 2 | 0.74 | 0.0194 | apoptosis |
| Destrin (DEST) | 5 | 0.75 | 0.0198 | actin regulation |
| Tubulin beta 1 (TBB1) | 11 | 0.88 | 0.0220 | Tubulin |
| Tubulin alpha 8 (TBA8) | 9 | 0.81 | 0.0248 | Tubulin |
| Clusterin-like 1 (CLUL1) | 2 | 0.76 | 0.0276 | apoptosis |
| Keratin, type II cytoskeletal 71 (K2C71) | 3 | 1.26 | 0.0284 | Keratin |
| 14-3-3 protein theta (1433T) | 21 | 0.92 | 0.0296 | 14-3-3 protein |
| Neuromodulin (NEUM) | 8 | 1.17 | 0.0319 | connectivity |
| Septin-7 (SEPT7) | 9 | 1.07 | 0.0335 | cell cycle |
| Sodium/potassium-transporting ATPase subunit alpha-3 (AT1A3) | 56 | 0.95 | 0.0349 | ATPase |
| Heat shock 70kDa 4 (HSP74) | 5 | 1.20 | 0.0361 | heat shock protein |
| BAG family molecular chaperone regulator 3 (BAG3) | 3 | 0.85 | 0.0361 | apoptosis |
| Synaptogyrin 3 (SNG3) | 6 | 1.21 | 0.0365 | synaptic vesicle |
| 14-3-3 protein epsilon (1433E) | 23 | 0.94 | 0.0406 | 14-3-3 protein |
| ATP synthase delta (ATPD) | 5 | 0.88 | 0.0411 | mitochondrion |
| Actin cytoplasmic 1 (ACTB) | 12 | 0.87 | 0.0414 | actin |
| Ras related Rab-3A (RAB3A) | 15 | 0.94 | 0.0435 | synaptic vesicles |
| Plasma membrane calcium-transporting ATPase 2 (AT2B2) | 32 | 0.93 | 0.0438 | ATPase |
| Stathmin (STMN1) | 4 | 1.07 | 0.0452 | neurogenesis |
| insulin-like growth factor-binding (ALS) | 2 | 0.74 | 0.0473 | cell adhesion |
| ***Hippocampus*** |  |  |  |  |
| FAD synthase (FAD1) | 2 | 0.68 | 0.0057 | FAD biosynthetic process |
| Protein argonaute-3 (AGO3) | 2 | 0.53 | 0.0074 | Translation |
| Visinin like protein 1 (VISL1) | 15 | 0.89 | 0.0077 | Rhodopsin |
| Glial fibrillary acidic protein (GFAP) | 28 | 1.08 | 0.0189 | cytoskeleton |
| Pyruvate kinase PKM (KPYM) | 54 | 0.94 | 0.0202 | Glycolysis |
| Macrophage migration inhibitory factor (MIF) | 3 | 0.78 | 0.0213 | inflammation |
| Testis-specific H1 histone | 4 | 0.91 | 0.0230 | Histone |
| ATP-dependent 6-phosphofructokinase, liver type (K6PL) | 2 | 0.78 | 0.0270 | glycolysis |
| Sodium/potassium-transporting ATPase subunit alpha-1 (AT1A1) | 48 | 0.96 | 0.0279 | ATPase |
| Voltage-dependent anion-selective channel protein 3 (VDAC3) | 8 | 1.06 | 0.0294 | mitochondrion |
| 60S ribosomal protein L6 (RL6) | 6 | 1.31 | 0.0302 | ribosome |
| Beta-arrestin 1 (ARRB1) | 2 | 0.73 | 0.0306 | protein transport |
| Ubiquitin-like modifier-activating enzyme 1 (UBA1) | 25 | 0.93 | 0.0310 | Ubiquitin |
| Heat shock 70kDa protein 1-like (HS71L) | 28 | 0.94 | 0.0316 | heat shock protein |
| 78 kDa glucose-regulated protein (GRP78) | 45 | 0.95 | 0.0335 | heat shock protein |
| Histone H1t (H1T) | 5 | 1.10 | 0.0340 | Histone |
| V type proton ATPase F (VATF) | 2 | 0.65 | 0.0359 | ATPase |
| Rho GTPase-activating 12 (RHG12) | 2 | 0.82 | 0.0390 | GTPase |
| BTB POZ domain containing protein KCTD12 (KCD12) | 3 | 1.13 | 0.0412 | neurotransmitter |
| L-lactate dehydrogenase B chain (LDHB) | 26 | 0.94 | 0.0435 | glycolysis |
| ATP-dependent 6-phosphofructokinase, platelet type (K6PP) | 5 | 0.86 | 0.0436 | glycolysis |
| Adenylate kinase isoenzyme 1 (KAD1) | 4 | 0.87 | 0.0444 | Energy |
| Heat shock-related 70kDa protein 2 (HSP72) | 48 | 1.03 | 0.0445 | heat shock protein |
| Myomesin-1 (MYOM1) | 3 | 0.87 | 0.0487 | cytoskeleton |
| Vesicular glutamate transporter 1 (VGLU1) | 3 | 1.10 | 0.0496 | neurotransmitter |
| ***Cerebellum*** |  |  |  |  |
| Sodium potassium transporting ATPase beta 2 (AT1B2) | 3 | 1.23 | 0.0052 | ATPase |
| Ras related Rab 8B (RAB8B) | 3 | 1.49 | 0.0078 | Synapse |
| Histone H2B type 1-F/J/L (H2B1F) | 4 | 0.91 | 0.0117 | Histone |
| Hexokinase-1 (HXK1) | 32 | 0.93 | 0.0136 | glycolysis |
| Histone H2B type 1-P (H2B1P) | 2 | 0.91 | 0.0186 | Histone |
| Guanine nucleotide binding G t alpha 3 (GNAT3) | 2 | 1.46 | 0.0193 | signalling |
| Cysteine and glycine-rich 1 (CSRP1) | 3 | 1.19 | 0.0195 | cytoskeleton |
| Microtubule-associated protein tau (TAU) | 5 | 0.91 | 0.0198 | cytoskeleton |
| 14-3-3 protein epsilon (1433E) | 21 | 0.93 | 0.0214 | 14-3-3 protein |
| Tripartite motif-containing 45 (TRI45) | 2 | 0.75 | 0.0350 | transcription |
| IQ calmodulin-binding motif-containing 1 (IQCB1) | 2 | 1.23 | 0.0386 | cytoskeleton |
| Putative adenosylhomocysteinase 2 (SAHH2) | 9 | 1.17 | 0.0387 | one-carbon metabolism |
| NADH dehydrogenase ubiquinone 1 alpha 10 (NDUAA) | 5 | 0.84 | 0.0393 | mitochondrion |
| Heat shock cognate 71 kDa protein (HSP7C) | 31 | 1.05 | 0.0400 | heat shock protein |
| Acyl-CoA-binding protein (ACBP) | 6 | 0.91 | 0.0407 | neurotransmitter |
| Excitatory amino acid transporter 1 (EAA1) | 9 | 0.81 | 0.0424 | neurotransmitter |
| Hippocalcin like 1 (HPCL1) | 3 | 0.81 | 0.0427 | rhodopsin |
| Cytochrome c oxidase 6B1 (CX6B1) | 5 | 1.14 | 0.0470 | mitochondrion |
| The table includes Uniprot ID, ratios (calculated based on average), and P values. Adjusted P values for the brain areas were respectively 0.4172 (FC), 0.9296 (HC), and 0.8302 (CB). | | | | |

| **Supplementary table S2. Proteins changed significantly in expression level using LC-MS^E^ on mouse frontal cortex (FC), hippocampus (HC) and cerebellum (CB)** | | | | |
| --- | --- | --- | --- | --- |
| **protein name** | **brain area** | **No. of peptides** | **ratio *Fmr1* KO/WT** | ***p*-value** |
| **synaptic proteins** |  |  |  |  |
| Excitatory amino acid transporter 1 (EAA1) | CB | 9 | 0.81 | 0.0424 |
| Vesicular glutamate transporter 1 (VGLU1) | HC | 3 | 1.1 | 0.0496 |
| BTB POZ domain containing protein KCTD12 (KCD12) | HC | 3 | 1.13 | 0.0412 |
| Succinate semialdehyde dehydrogenase (SSDH) | FC | 2 | 1.16 | 0.0135 |
| Neuromodulin (NEUM) | FC | 8 | 1.17 | 0.0319 |
| Cysteine and glycine-rich 1 (CSRP1) | CB | 3 | 1.19 | 0.0195 |
| Synaptogyrin 3 (SNG3) | FC | 6 | 1.21 | 0.0365 |
| Guanine nucleotide binding G t alpha 3 (GNAT3) | CB | 2 | 1.46 | 0.0193 |
| Ras related Rab 8B (RAB8B) | CB | 3 | 1.49 | 0.0078 |
| **ATPase** |  |  |  |  |
| V type proton ATPase F (VATF) | HC | 2 | 0.65 | 0.0359 |
| Sarcoplasmic endoplasmic reticulum calcium ATPase 2 (AT2A2) | FC | 4 | 0.84 | 0.0059 |
| Sodium potassium transporting ATPase beta 2 (AT1B2) | CB | 3 | 1.23 | 0.0052 |
| **transcription** |  |  |  |  |
| Tripartite motif-containing 45 (TRI45) | CB | 2 | 0.75 | 0.035 |
| lysine-specific histone demethylase 1A (KDM1A) | FC | 2 | 0.86 | 0.0109 |
| histone H1t (H1T) | HC | 5 | 1.1 | 0.034 |
| **translation** |  |  |  |  |
| Protein argonaute-3 (AGO3) | HC | 2 | 0.53 | 0.0074 |
| Ubiquitin 60S ribosomal L40 (RL40) | FC | 13 | 0.87 | 0.0104 |
| 60S ribosomal protein L6 (RL6) | HC | 6 | 1.31 | 0.0302 |
| **transport** |  |  |  |  |
| beta-arrestin 1 (ARRB1) | HC | 2 | 0.73 | 0.0306 |
| Ras related Rab 43 (RAB43) | FC | 10 | 0.82 | 0.0115 |
| **rhodopsin regulation** |  |  |  |  |
| Hippocalcin like 4 (HPCL4) | FC | 3 | 0.74 | 0.0182 |
| Hippocalcin like 1 (HPCL1) | CB | 3 | 0.81 | 0.0427 |
| Visinin like protein 1 (VISL1) | HC | 15 | 0.89 | 0.0077 |
| **Mitochondrial/metabolic proteins** |  |  |  |  |
| Cytochrome c oxidase 5A (COX5A) | FC | 5 | 0.73 | 0.0112 |
| 6 phosphofructokinase liver (K6PL) | HC | 2 | 0.78 | 0.027 |
| complex I intermediate-associated 30 (CIA30) | FC | 4 | 0.82 | 0.0115 |
| NADH dehydrogenase ubiquinone 1 alpha 10 (NDUAA) | CB | 5 | 0.84 | 0.0393 |
| 6 phosphofructokinase (K6PP) | HC | 5 | 0.86 | 0.0436 |
| ATP synthase delta (ATPD) | FC | 5 | 0.88 | 0.0411 |
| Cytochrome c oxidase 6B1 (CX6B1) | CB | 5 | 1.14 | 0.047 |
| The table includes Uniprot ID, ratios (calculated based on average), and P values. Adjusted P values for the brain areas were respectively 0.4172 (FC), 0.9296 (HC), and 0.8302 (CB). Significant proteins with an increase/decrease greater than 10% are depicted here. FC = frontal cortex; HC = hippocampus; CB = cerebellum | | | | |

| **Supplementary table S3. Analysis of protein levels in mouse brain synaptosome fractions of Fmr1 KO and WT** | | | |
| --- | --- | --- | --- |
| **protein name** | **No. of peptides** | **ratio *Fmr1* KO/WT** | ***p*-value** |
| ***Hippocampus*** |  |  |  |
| NADH dehydrogenase ubiquinone flavoprotein 1 (NDUV1) | 32 | 0.89 | 0.0006 |
| Uridine 5 monophosphate synthase (UMPS) | 2 | 0.82 | 0.0010 |
| Septin 10 (SEP10) | 5 | 0.57 | 0.0011 |
| Guanine nucleotide binding protein G i alpha 1 (GNAI1) | 21 | 0.90 | 0.0016 |
| Calmodulin protein 3 (CALL3) | 10 | 1.18 | 0.0034 |
| cytoplasmic FMR1-interacting protein 2 (CYFP2) | 19 | 1.08 | 0.0051 |
| Septin 11 (SEP11) | 27 | 1.06 | 0.0066 |
| Glucose-induced degradation 8 homolog (GID8) | 2 | 1.11 | 0.0074 |
| Guanine nucleotide binding protein G s alpha Xlas (GNAS1) | 10 | 1.12 | 0.0104 |
| Estradiol 17 beta dehydrogenase 8 (DHB8) | 2 | 0.78 | 0.0113 |
| Neutral amino acid transporter A (SATT) | 3 | 1.10 | 0.0116 |
| Tetraspanin 7 (TSN7) | 2 | 1.13 | 0.0139 |
| V-type proton ATPase subunit C 1 (VATC1) | 32 | 1.05 | 0.0143 |
| Tyrosine phosphatase non receptor substrate 1 (SHPS1) | 22 | 0.89 | 0.0148 |
| Leucine-rich repeat-containing 8D (LRC8D) | 2 | 1.35 | 0.0149 |
| Peroxiredoxin-6 (PRDX6) | 27 | 0.93 | 0.0184 |
| Glutamate decarboxylase 1 (DCE1) | 6 | 1.12 | 0.0187 |
| Hippocalcin like 1 (HPCL1) | 6 | 0.88 | 0.0193 |
| Pikachurin (EGFLA) | 2 | 0.89 | 0.0203 |
| Dihydrolipoyllysine succinyltransferase component (ODO2) | 16 | 0.85 | 0.0207 |
| Homeobox protein Hox C9 (HXC9) | 2 | 1.13 | 0.0244 |
| Neuronal-specific septin 3 (SEPT3) | 26 | 1.07 | 0.0253 |
| Cofilin-1 (COF1) | 23 | 1.04 | 0.0269 |
| ATP synthase subunit beta, mitochondrial (ATPB) | 50 | 0.93 | 0.0282 |
| Glutamate receptor 4 (GRIA4) | 3 | 1.19 | 0.0288 |
| Bicaudal D related protein 2 (BICR2) | 3 | 1.14 | 0.0295 |
| 26S protease regulatory subunit 8 (PRS8) | 3 | 0.69 | 0.0340 |
| Neuron specific calcium binding hippocalcin (HPCA) | 16 | 0.90 | 0.0343 |
| Heat shock 70 kDa protein 1A (HS71A) | 34 | 0.95 | 0.0364 |
| NADH dehydrogenase [ubiquinone] 1 alpha subcomplex subunit 10, mitochondrial (NDUAA) | 28 | 0.91 | 0.0377 |
| Meiosis-specific with OB domain-containing (MEIOB) | 3 | 0.85 | 0.0379 |
| Intercellular adhesion molecule 5 (ICAM5) | 34 | 0.92 | 0.0390 |
| Rho GTPase activating protein 1 (RHG01) | 2 | 1.16 | 0.0466 |
| Immunoglobulin superfamily member 8 (IGSF8) | 15 | 0.94 | 0.0467 |
| ***Cerebellum*** |  |  |  |
| Neuronal growth regulator 1 (NEGR1) | 12 | 1.07 | 0.0038 |
| Ribonuclease UK114 (UK114) | 7 | 0.92 | 0.0047 |
| Heat shock 70 kDa 1A (HS71A) | 21 | 1.12 | 0.0048 |
| Glycerol 3 phosphate dehydrogenase NAD cytoplasmic (GPDA) | 34 | 0.95 | 0.0049 |
| Serine threonine protein phosphatase 2A 65 kDa regulatory subunit A alpha isoform (2AAA) | 23 | 0.92 | 0.0062 |
| Myristoylated alanine rich C kinase substrate (MARCS) | 16 | 0.91 | 0.0085 |
| Transitional endoplasmic reticulum ATPase (TERA) | 33 | 0.92 | 0.0093 |
| Pyruvate kinase isozymes (KPYM) | 53 | 0.96 | 0.0099 |
| Plakophilin-1 (PKP1) | 3 | 0.89 | 0.0109 |
| Ras related Rab 3C (RAB3C) | 20 | 0.88 | 0.0119 |
| Ras related protein Rab 3D (RAB3D) | 15 | 0.93 | 0.0120 |
| Synaptic vesicle glycoprotein 2B (SV2B) | 8 | 0.87 | 0.0124 |
| 6 phosphofructokinase muscle type (K6PF) | 52 | 0.94 | 0.0154 |
| cAMP dependent protein kinase type II alpha regulatory subunit (KAP2) | 5 | 1.08 | 0.0201 |
| F actin capping protein alpha 2 (CAZA2) | 3 | 0.90 | 0.0209 |
| ADP ribosylation factor 4 (ARF4) | 7 | 1.06 | 0.0213 |
| Nucleoside diphosphate kinase B (NDKB) | 3 | 0.92 | 0.0219 |
| Profilin 1 (PROF1) | 5 | 0.91 | 0.0223 |
| L-asparaginase (ASGL1) | 10 | 0.93 | 0.0263 |
| Homeobox protein Hox A9 (HXA9) | 2 | 0.82 | 0.0273 |
| Adenosine deaminase-like (ADAL) | 2 | 0.87 | 0.0275 |
| Guanine nucleotide binding protein Gi Gs Gt subunit beta 2 (GBB2) | 15 | 1.06 | 0.0296 |
| Myosin regulatory light chain 2 (MLRV) | 2 | 0.83 | 0.0319 |
| Guanine nucleotide binding protein G t subunit alpha 2 (GNAT2) | 12 | 0.93 | 0.0325 |
| Cytosolic 10 formyltetrahydrofolate dehydrogenase (AL1L1) | 25 | 0.95 | 0.0352 |
| Guanine nucleotide binding G I G S G T beta 3 (GBB3) | 12 | 0.90 | 0.0353 |
| Sulfated glycoprotein 1 (SAP) | 8 | 0.72 | 0.0369 |
| Sarcoplasmic endoplasmic reticulum calcium ATPase 2 (AT2A2) | 55 | 0.97 | 0.0371 |
| Tropomodulin-2 (TMOD2) | 4 | 0.90 | 0.0400 |
| Protein SERAC1 (SRAC1) | 4 | 0.88 | 0.0414 |
| BSD domain-containing 1 (BSDC1) | 3 | 0.86 | 0.0432 |
| NADH ubiquinone oxidoreductase chain 4 (NU4M) | 8 | 1.10 | 0.0435 |
| Dihydropyrimidinase related protein 4 (DPYL4) | 17 | 1.06 | 0.0486 |
| The table includes Uniprot ID, ratios (calculated based on average), and P values. Adjusted P values for the brain areas were respectively 0.95318 (HC) and 0.91602 (CB). | | | |

| **Supplementary table S4. Proteins significantly changed in expression level using LC-MS^E^ with mouse brain synaptosome fractions from the hippocampus (HC) and cerebellum (CB)** | | | | |
| --- | --- | --- | --- | --- |
| **protein name** | **synaptosome fraction** | **No. of peptides** | **ratio *Fmr1* KO/WT** | ***p*-value** |
| **cell signalling** |  |  |  |  |
| Guanine nucleotide binding protein Gi alpha 1 (GNAI1) | HC | 21 | 0.90 | 0.0016 |
| Guanine nucleotide binding protein Gs alpha Xlas (GNAS1) | HC | 10 | 1.12 | 0.0104 |
| Leucine-rich repeat-containing 8D (LRC8D) | HC | 2 | 1.35 | 0.0149 |
| Rho GTPase activating protein 1 (RHG01) | HC | 2 | 1.16 | 0.0466 |
| Guanine nucleotide binding G(I)/G(S)/G(T) beta-3 (GBB3) | CB | 12 | 0.90 | 0.0353 |
| Hippocalcin like 1 (HPCL1) | HC | 6 | 0.88 | 0.0193 |
| Sulfated glycoprotein 1 (SAP) | CB | 8 | 0.72 | 0.0369 |
| **synaptic signalling** |  |  |  |  |
| Glutamate decarboxylase 1 (DCE1) | HC | 6 | 1.12 | 0.0187 |
| Glutamate receptor 4 (GRIA4) | HC | 3 | 1.19 | 0.0288 |
| Ras related Rab 3C (RAB3C) | CB | 20 | 0.88 | 0.0119 |
| Synaptic vesicle glycoprotein 2B (SV2B) | CB | 8 | 0.87 | 0.0124 |
| **connectivity** |  |  |  |  |
| Tyrosine phosphatase non receptor substrate 1 (SHPS1) | HC | 22 | 0.89 | 0.0148 |
| Plakophilin-1 (PKP1) | CB | 3 | 0.89 | 0.0109 |
| **neuron development** |  |  |  |  |
| Septin 10 (SEP10) | HC | 5 | 0.57 | 0.0011 |
| Tetraspanin 7 (TSN7) | HC | 2 | 1.13 | 0.0139 |
| Pikachurin (EGFLA) | HC | 2 | 0.89 | 0.0203 |
| Meiosis-specific with OB domain-containing (MEIOB) | HC | 3 | 0.85 | 0.0379 |
| **mitochondrial/metabolic proteins** |  |  |  |  |
| NADH dehydrogenase ubiquinone flavoprotein 1 (NDUV1) | HC | 32 | 0.89 | 0.0006 |
| Estradiol 17 beta dehydrogenase 8 (DHB8) | HC | 2 | 0.78 | 0.0113 |
| Dihydrolipoyllysine succinyltransferase component (ODO2) | HC | 16 | 0.85 | 0.0207 |
| Protein SERAC1 (SRAC1) | CB | 4 | 0.88 | 0.0414 |
| NADH ubiquinone oxidoreductase chain 4 (NU4M) | CB | 8 | 1.10 | 0.0435 |
| **transcription** |  |  |  |  |
| Uridine 5 monophosphate synthase (UMPS) | HC | 2 | 0.82 | 0.0010 |
| Homeobox protein Hox C9 (HXC9) | HC | 2 | 1.13 | 0.0244 |
| 26S protease regulatory subunit 8 (PRS8) | HC | 3 | 0.69 | 0.0340 |
| Homeobox protein Hox A9 (HXA9) | CB | 2 | 0.82 | 0.0273 |
| Adenosine deaminase-like (ADAL) | CB | 2 | 0.87 | 0.0275 |
| The table includes Uniprot ID, ratios (calculated based on average), and P values. Adjusted P values for the synaptic fractions were respectively 0.9532 (HC) and 0.9960 (CB). Significant proteins with an increase/decrease greater than 10% are depicted here. HC = hippocampus; CB = cerebellum | | | | |

## GO-enrichment analysis

GO-enrichment analysis was performed to identify altered networks in brain tissue and synaptosome fractions of the *Fmr1* KO mouse model using all significantly changed proteins with a ratio *Fmr1* KO/WT of >10%. Protein-Protein Interaction (PPI) networks were constructed from the significantly changed proteins by finding first-degree interacting neighbours across 3 databases: MINT, IntAct and Uniprot. Enrichment was computed on each network using the ClueGO package in Cytoscape[4]. Proteins were annotated according to the Mus Musculus gene ontology database (ontology: Biological Process), which creates a functionally organized GO-pathway term network. A two-sided hypergeometric distribution was used to compute the statistical significance of each GO annotation, describing the probabilities associated with sampling randomly without replacement from a finite network of proteins where all proteins have an equal chance of being drawn. Only GO terms with a significant q value (q <0.05, Benjamini-Hochberg test) were taken forward. Subsequently, fuzzy heuristic partitioning was used to enhance the biological interpretation of the pathways[5] and a diffusion-type manifold embedding technique was used to emphasize short range interactions between GO terms[6]. For each GO term of interest, similar terms were represented by positive distances while dissimilar terms were represented by negative distances. This allowed functional groups of similar terms to be created for each tissue sample (**supplementary table S5**) and synaptosome (**supplementary table S6**). The functional correlation between synaptosomes and tissue was examined by constructing a heatmap showing the percentage overlap of GO terms between the functional groups (**supplementary figure S2**).

| **Supplementary table S5. GO term enrichment of mouse brain tissue of Fmr1 KO and WT** | | | |
| --- | --- | --- | --- |
| **GO terms** | ***q*-value** | **proteins** | **significant protein (1), neighbour (0)** |
| **Frontal cortex** | | | |
| protein polymerization | 1.49*10^-9^ | Micall2, Pfn1, Pfn2, Prkce, Sept2, Tmod3, Tuba1a, Tuba8, Tubb4b, Tubb5 | 1 |
| actin polymerization or depolymerization | 5.86*10^-8^ | Cfl2, Dstn, Micall2, Pfn1, Pfn2, Plekhh2, Prkce, Tmod3 | 1 |
| regulation of actin filament depolymerization | 4.11*10^-5^ | Cfl2, Dstn, Plekhh2, Tmod3 | 1 |
| cellular potassium ion homeostasis | 0.0002 | Camk2d, Kcnma1 | 1 |
| negative regulation of histone H3-K4 methylation | 0.0002 | Gfi1b, Kdm1a | 1 |
| regulation of cell projection assembly | 0.0002 | Espn, Gap43, Pfn1, Pfn2 | 1 |
| positive regulation of actin filament depolymerization | 0.0004 | Cfl2, Dstn | 1 |
| regulation of histone H3-K9 methylation | 0.0007 | Gfi1b, Kdm1a | 1 |
| regulation of neural precursor cell proliferation | 0.0015 | Kdm1a, Kdm2b, Lrrk2 | 1 |
| histone demethylation | 0.0025 | Kdm1a, Kdm2b | 1 |
| positive regulation of Ras protein signal transduction | 0.0033 | Camk2d, Csf1 | 1 |
| regulation of filopodium assembly | 0.0036 | Espn, Gap43 | 1 |
| negative regulation of cytoskeleton organization | 4.71*10^-5^ | Espn, Pfn1, Pfn2, Plekhh2, Tmod3 | 0 |
| dendrite morphogenesis | 5.32*10^-5^ | Dlg4, Grin1, Ilk, Lrrk2, Ywhah | 0 |
| exploration behavior | 6.83*10^-5^ | Dlg4, Lrrk2, Prkce | 0 |
| actin filament polymerization | 7.52*10^-5^ | Micall2, Pfn1, Pfn2, Prkce, Tmod3 | 0 |
| negative regulation of protein dephosphorylation | 0.0002 | Ywhab, Ywhae | 0 |
| regulation of long-term neuronal synaptic plasticity | 0.0003 | Dlg4, Grin1, Grin2b | 0 |
| membrane depolarization | 0.0003 | Dlg4, Grin1, Grin2b, Ywhah | 0 |
| negative regulation of actin filament polymerization | 0.0004 | Pfn1, Pfn2, Tmod3 | 0 |
| response to ammonium ion | 0.0004 | Dlg4, Grin1, Prkce | 0 |
| lateral ventricle development | 0.0004 | Kdm2b, Rpgrip1l | 0 |
| histone H2A monoubiquitination | 0.0004 | Kdm2b, Rnf2 | 0 |
| endodermal cell fate specification | 0.0005 | Nanog, Pou5f1 | 0 |
| regulation of ruffle assembly | 0.0007 | Pfn1, Pfn2 | 0 |
| positive regulation of actin filament polymerization | 0.0007 | Pfn1, Pfn2, Prkce | 0 |
| determination of adult lifespan | 0.0011 | Lrrk2, Tfcp2l1 | 0 |
| regulation of cell-matrix adhesion | 0.0012 | Cask, Csf1, Ilk | 0 |
| glutamate receptor signaling pathway | 0.0012 | Dlg4, Grin1, Grin2b | 0 |
| mesodermal cell fate commitment | 0.0017 | Nanog, Pou5f1 | 0 |
| regulation of cell fate specification | 0.0018 | Nanog, Pou5f1 | 0 |
| dendritic spine morphogenesis | 0.0028 | Dlg4, Lrrk2 | 0 |
| negative regulation of actin filament depolymerization | 0.0037 | Plekhh2, Tmod3 | 0 |
| positive regulation of stress fiber assembly | 0.0037 | Pfn1, Pfn2 | 0 |
| olfactory bulb development | 0.0053 | Lrrk2, Rpgrip1l | 0 |
| **Hippocampus** | | | |
| negative regulation of cell cycle arrest | 2.58*10^-6^ | Cdk9, Mdm2, Mif | 1 |
| negative regulation of DNA damage response, signal transduction by p53 class mediator | 0.0001 | Mdm2, Mif | 1 |
| ATP hydrolysis coupled proton transport | 0.0005 | Atp6v1a, Atp6v1f | 1 |
| positive regulation of Ras protein signal transduction | 0.0005 | Arrb1, Csf1 | 1 |
| negative regulation of protein dephosphorylation | 4.10*10^-5^ | Ywhab, Ywhae | 0 |
| intermediate filament organization | 0.0005 | Dnajb6, Vim | 0 |
| **Cerebellum** | | | |
| ATP catabolic process | 2.31*10^-5^ | Atp1b2, Atp2a2, Atp5a1, Hsp90aa1, Hspa8, Myh10, Myh9, Myo1c | 1 |
| neuromuscular process controlling balance | 6.58*10^-5^ | Dlg4, Hexa, Kcnma1, Myh10, Slc1a3 | 1 |
| glutamate biosynthetic process | 0.0007 | Glud1, Slc1a3 | 1 |
| mitochondrial transport | 0.0008 | Acaa2, Hsp90aa1, Hspa4, Slc1a3, Ywhaz | 1 |
| protein folding | 4.50*10^-7^ | Cct3, Cct6a, Dnaja1, Dnaja3, Hsp90aa1, Hsp90ab1, Hspa8, Hspa9, Hsph1 | 0 |
| negative regulation of protein dephosphorylation | 0.0007 | Ywhab, Ywhae | 0 |
| cellular response to interleukin-4 | 0.0007 | Hsp90ab1, Hspa5, Tuba1b | 0 |
| positive regulation of cell migration by vascular endothelial growth factor signalling pathway | 0.0008 | Hspb1, Myo1c | 0 |
| cell junction assembly | 0.0008 | Actn4, Epb4.1l5, Jup, Myo1c, Plec | 0 |
| response to unfolded protein | 0.0008 | Hsp90aa1, Hspa5, Hspb1, Hsph1 | 0 |
| protein targeting to mitochondrion | 0.0011 | Hsp90aa1, Hspa4, Ywhaz | 0 |
| lateral ventricle development | 0.0012 | Myh10, Rpgrip1l | 0 |
| cell-cell junction assembly | 0.0026 | Actn4, Jup, Myo1c | 0 |
| negative regulation of inflammatory response to antigenic stimulus | 0.0026 | Psma1, Psmb4 | 0 |
| iron-sulfur cluster assembly | 0.0026 | Ciao1, Mms19 | 0 |
| positive regulation of protein import into nucleus, translocation | 0.0026 | Hsp90aa1, Hsp90ab1 | 0 |
| chaperone mediated protein folding requiring cofactor | 0.0033 | Hspa8, Hsph1 | 0 |
| mitochondrial membrane organization | 0.0057 | Acaa2, Hsp90aa1, Hspa4 | 0 |
| cerebellum development | 0.0065 | Hspa5, Myh10, Rpgrip1l | 0 |
| cerebellar Purkinje cell layer development | 0.0085 | Hspa5, Myh10 | 0 |
| vesicle docking | 0.0092 | Exoc4, Exoc5 | 0 |
| Golgi to plasma membrane transport | 0.0112 | Arfgef2, Sptbn1 | 0 |
| binding of sperm to zona pellucida | 0.0119 | Cct3, Cct6a | 0 |
| regulation of cell junction assembly | 0.0139 | Epb4.1l5, Myo1c | 0 |
| Enriched networks indicated with "1" are pathways including at least one significant protein, "0" indicates the nearest neighbour using the selected significant proteins | | | |

| **Supplementary table S6. GO term enrichment of mouse hippocampus and cerebellum synaptosome fraction** | | | |
| --- | --- | --- | --- |
| **GO Terms** | **q-value** | **proteins** | **significant protein (1), neighbour (0)** |
| **Hippocampus** | | | |
| negative regulation of protein dephosphorylation | 1.86*10^-5^ | Ywhab, Ywhae | 0 |
| glutamate receptor signalling pathway | 3.37*10^-5^ | Dlg4, Gria2, Gria4 | 1 |
| **Cerebellum** | | | |
| exploration behaviour | 1.53*10^-6^ | Dlg4, Lrrk2, Prkce | 0 |
| negative regulation of protein dephosphorylation | 1.28*10^-5^ | Ywhab, Ywhae | 0 |
| dendritic spine morphogenesis | 0.0003 | Dlg4, Lrrk2 | 0 |
| positive regulation of proteasomal ubiquitin-dependent protein catabolic process | 0.0006 | Lrrk2, Vcp | 0 |
| Enriched networks indicated with "1" are pathways including at least one significant protein, "0" indicates the nearest neighbour using the selected significant proteins | | | |


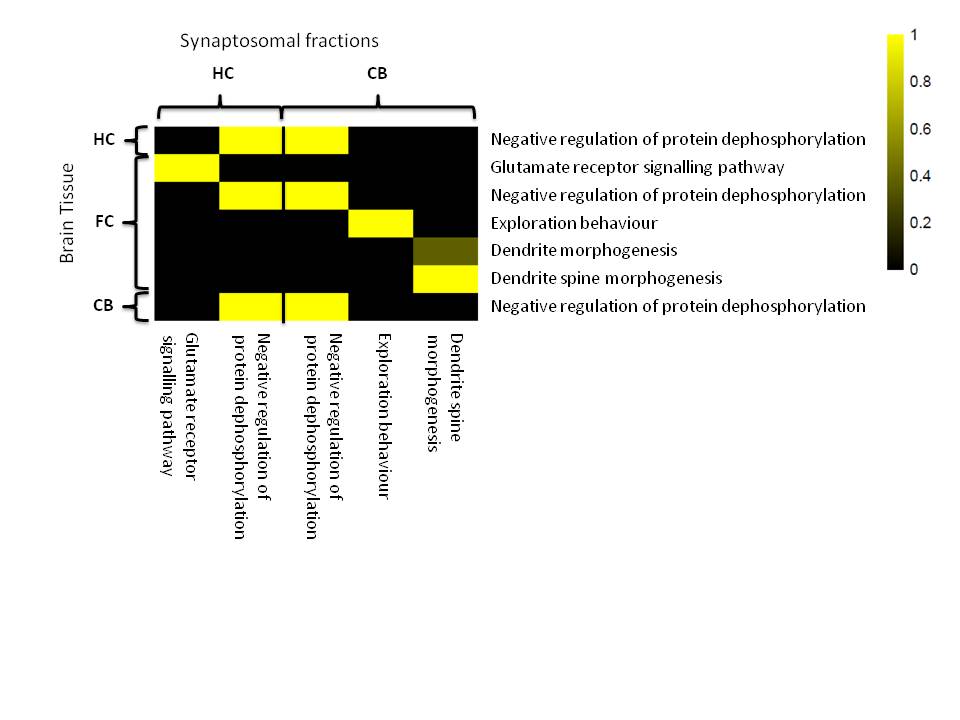


**Supplementary figure S2. Overlapping significant GO-enriched networks between Fmr1 KO mouse brain tissue and synaptosome fractions.** The gradual change in yellow colour indicates the ratio of overlap between the identified networks. Negative regulation of protein dephosphorylation was indicated in all studies. Other overlapping networks are involved in synaptic signalling and exploration behaviour.

## Primary hippocampal and cerebellar neuron culture

To study synaptic vesicle dynamics in live cell imaging, primary hippocampal and cerebellar neurons were cultured. The dissected mouse brains were placed in ice cold Hank’s Balanced Salt Solution (HBSS, Life Technologies). After dissection, the hippocampi and cerebella were dissociated as discussed in [7] using papain and mechanical treatment for 30 minutes at 34 degrees. The neurons were extracted in a stepwise manner by first adding HBSS (8mL) and 10% fetal bovine serum (2mL, FBS, Life Technologies), which is followed by centrifugation for 4 minutes at 200G. The supernatant is supplemented with HBSS (5mL), MgSO4 (4mM) and DNase (10μL, Roche), and the cells are dissociated using Nylon Net Filters (180μm NY8H, Merck Millipore Ltd.). Removing of DNase occurred by washing with HBSS and centrifugation for 2x4 minutes at 200 G. Respective media were as follows: cerebella: Primary Neuron Basal Medium (PNBM, 50mL, Lonza), insulin (20μg/mL), Gentamicin 2000x (5μg/mL, Life Technologies), GlutaMAX (100x, Life Technologies), progesterone (40nM, Sigma), triiodo-L-Thyronine (0.5ng/mL, Sigma), albumin (100μg/mL), B-27 supplements (50x, Life Technologies), apo-transferin (200μg/mL, Sigma); hippocampi: Neurobasal medium (NB, 50mL, Life Technologies), Gentamicin 2000x (5μg/mL, Life Technologies), GlutaMAX (100x, Life Technologies), B-27 supplements (50x, Life Technologies). Per well in respective media with 10% FBS, a concentration of 5x10^6^ cells for cerebellum and 1x10^6^ for hippocampus were plated on ⌀19mm coverslips coated with poly-D-lysine (200μg/mL, Sigma). After 1.5 hour the medium+FBS was replaced with medium only. After 3 days *in vitro*, ara-C (2μM) was added to the cerebellum cells to inhibit glia growth.

## Visualization of synaptic bouton activity with live cell imaging

This part of the supplementary information describes the detailed process of the visualization of synaptic bouton activity using live cell imaging. Visualization of synaptic bouton activity is based on the uptake and unloading of styryl FM1-43 dye (Molecular Probes, Life Technologies) by synaptic vesicles in the primary hippocampal or cerebellar neurons. The primary neurons were plated on coverslips and incubated for 10 minutes with pre-warmed low K^+^ medium (5mM KCl) and subsequently loaded with FM1-43 styryl dye (5μg/mL, Molecular Probes, Life Technologies) for 5 minutes in high-K^+^ medium (50mM KCl in 10mL respective medium) (**Supplementary figure S3**). To remove surface bound dye, the neurons were washed for 10 minutes with respective medium. Before stimulation of the synaptic boutons, baseline measurements were recorded for 100 seconds, which was followed by recordings of dye unloading kinetics via stimulation of the primary neurons with high-K^+^ medium. Live cell images were acquired of synaptic bouton activity using spinning-disk confocal microscopy (SDCM) technique based on a Nikon Eclipse Ti-E inverted microscope with a 40x 1.30 NA oil objective (Plan Fluor DIC, Nikon) connected to a Yokogawa dual spinning-disk confocal scanner unit (Yokogawa, CSU-X1) with a 630/75m bandpass emission filter (Chroma). FM1-43 was excited with a 491nm 50mW diode pumped solid state laser (Calypso, Cobolt), which caused emission at 566nm. Images were recorded with an EMCCD camera (QuantEM:512C, Photometrics) at a magnification of 164 nm/pixel. The microscope was equipped with a perfect focus system (Nikon). An incubation chamber (zylxs type INUG2, Tokai Hit) was used to keep the cells at 37°C and 5% CO_2_ in a humidified environment. Timelapse imaging was performed for 20 minutes with 10 second intervals. The microscope setup was controlled by MetaMorph *7.7* software (Molecular Devices).


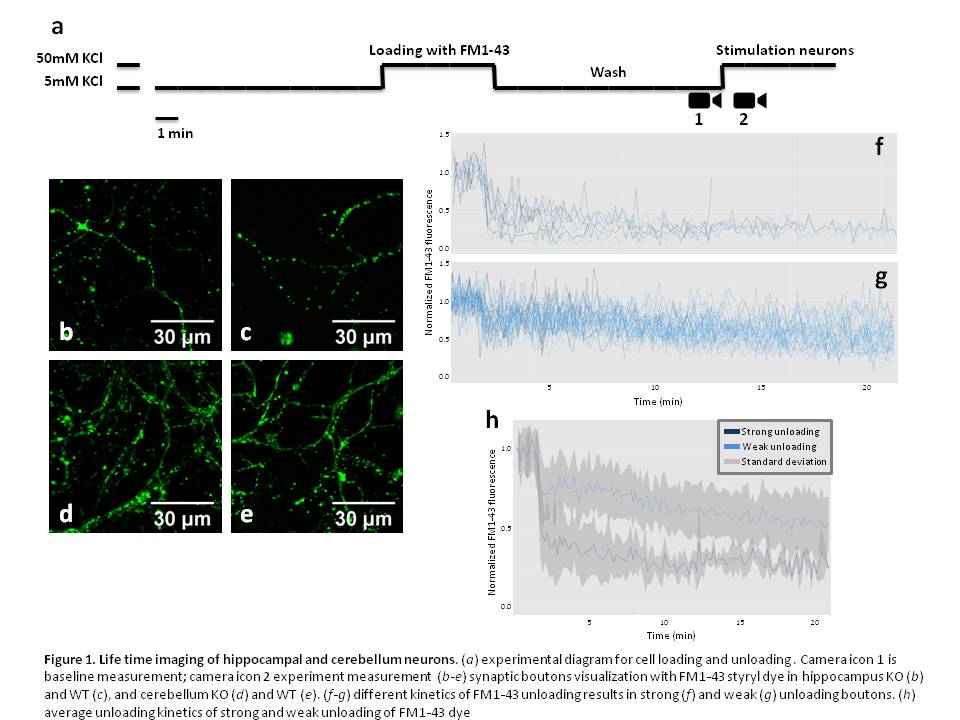


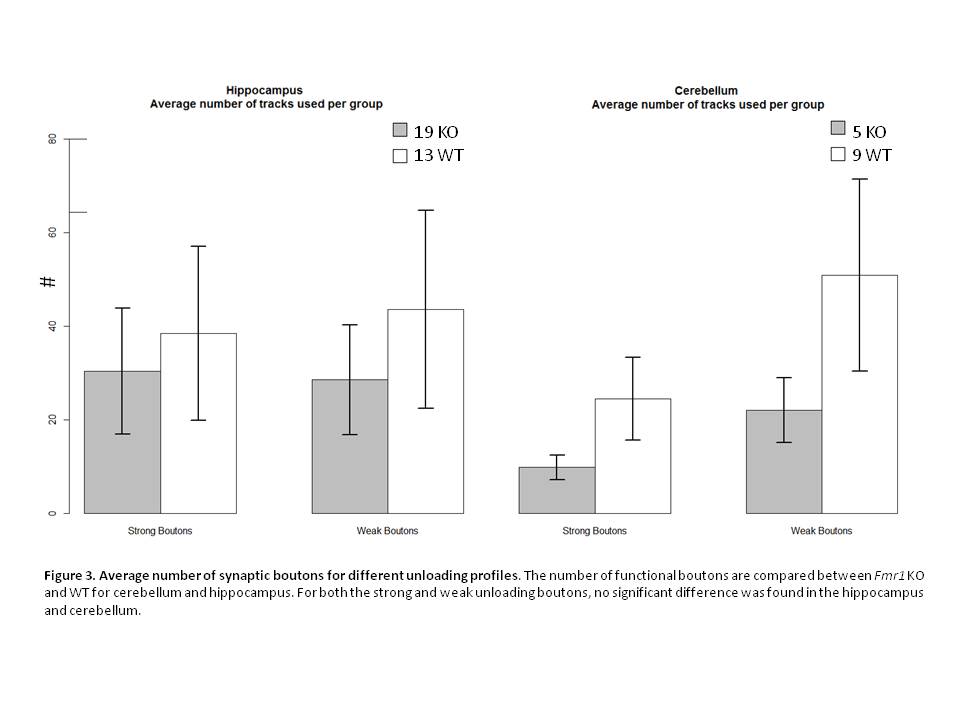


**Supplementary figure S3. Live cell imaging of hippocampal and cerebellar neurons**. (a) Experimental diagram for cell loading and unloading. Camera icon 1 depicts a representative baseline measurement; camera icon 2 a representative experimental measurement; (b-e) synaptic bouton visualization with FM1-43 styryl dye in hippocampus KO (b) and WT (c), and cerebellum KO (d) and WT (e). (f-g) different kinetics of FM1-43 unloading results in strong (f) and weak (g) unloading boutons. (h) Average unloading kinetics of strong and weak unloading of FM1-43 dye.

**Supplementary figure S4. Average number of synaptic boutons for different unloading profiles**. The number of functional boutons was compared between Fmr1 KO and WT for cerebellum and hippocampus. The number of hippocampal neurons investigated is greater than the number of cerebellar neurons, due to the difficulty of culturing cerebellar neurons. For both the strong and weak unloading boutons, no significant differences were found in hippocampus or cerebellum. Data represent the mean ± s.e.m.

## Ultrastructure analysis using electron microscopy

This part describes the ultrastructural analysis performed using electron microscopy to investigate subcellular changes in synapses. Cerebellar tissue was obtained from *Fmr1* KO mice, which were anesthetized (Nembutal, 50mg/kg) and subsequently perfused transcardially with 4% paraformaldehyde and 0.5% glutaraldehyde in cacodylate buffer. Further procedures were performed according to Hoebeek, 2008 [8]. The brains were cut into 80 μm thick coronal sections with a vibratome and calbindin immunocytochemistry was performed by incubating the sections with rabbit anti-calbindin antibody and diaminobenzidine for visualization of Purkinje cells. The sections were osmicated with 2% osmium in 8% glucose solution, dehydrated in dimethoxypropane and stained en block with 3% uranyl acetate/70% ethanol for 60 minutes and embedded in Araldite (Durcupan, Fluka, Germany). Ultrathin sections (70-90 nm) were cut using an Ultramicrotome (Leica, Germany), mounted on copper grids, and counterstained with uranyl acetate and lead citrate [8]. Purkinje cell terminals were recorded at magnifications x4900, x7000, x9800 and analyzed under the electron microscope (Philips, Eindhoven, the Netherlands). The digital electron micrographs were analyzed for total vesicle number, active zone (AZ) length, presynaptic area, and vesicle density (nm^2^) using Fiji, ImageJ v2.0.0[9]. The observer was blind to genotype during the procedure. The data were statistical analyzed using a t-test with permutations (AZ length, presynaptic area, and vesicle density) and correlation (density with AZ length) in R statistical programming language (version 3.1.1 [10]).

## Supplemental References

1. Broek JA, Guest PC, Rahmoune H, Bahn S: **Proteomic analysis of post mortem brain tissue from autism patients: evidence for opposite changes in prefrontal cortex and cerebellum in synaptic connectivity-related proteins.** *Mol Autism* 2014, **5:**41.

2. Wesseling H, Want EJ, Guest PC, Rahmoune H, Holmes E, Bahn S: **Hippocampal proteomic and metabonomic abnormalities in neurotransmission, oxidative stress and apoptotic pathways in a chronic phencyclidine rat model.** *J Proteome Res* 2015.

3. Krishnamurthy D, Harris LW, Levin Y, Koutroukides TA, Rahmoune H, Pietsch S, Vanattou-Saifoudine N, Leweke FM, Guest PC, Bahn S: **Metabolic, hormonal and stress-related molecular changes in post-mortem pituitary glands from schizophrenia subjects.** *World J Biol Psychiatry* 2013, **14:**478-489.

4. Bindea G, Mlecnik B, Hackl H, Charoentong P, Tosolini M, Kirilovsky A, Fridman WH, Pages F, Trajanoski Z, Galon J: **ClueGO: a Cytoscape plug-in to decipher functionally grouped gene ontology and pathway annotation networks.** *Bioinformatics* 2009, **25:**1091-1093.

5. Frohlich H, Speer N, Poustka A, Beissbarth T: **GOSim--an R-package for computation of information theoretic GO similarities between terms and gene products.** *BMC Bioinformatics* 2007, **8:**166.

6. Lerman G, Shakhnovich BE: **Defining functional distance using manifold embeddings of gene ontology annotations.** *Proc Natl Acad Sci U S A* 2007, **104:**11334-11339.

7. Tabata T, Sawada S, Araki K, Bono Y, Furuya S, Kano M: **A reliable method for culture of dissociated mouse cerebellar cells enriched for Purkinje neurons.** *J Neurosci Methods* 2000, **104:**45-53.

8. Hoebeek FE, Khosrovani S, Witter L, De Zeeuw CI: **Purkinje cell input to cerebellar nuclei in tottering: ultrastructure and physiology.** *Cerebellum* 2008, **7:**547-558.

9. Schindelin J, Arganda-Carreras I, Frise E, Kaynig V, Longair M, Pietzsch T, Preibisch S, Rueden C, Saalfeld S, Schmid B, et al: **Fiji: an open-source platform for biological-image analysis.** *Nat Methods* 2012, **9:**676-682.

10. R ct: *R: A language and environment for statistical computing.* Vienna, Austria; 2013.
